# From intra- to extra-uterine: early phase design of a transfer to extra-uterine life support through medical simulation

**DOI:** 10.3389/fmedt.2024.1371447

**Published:** 2024-08-20

**Authors:** J. S. van Haren, F. L. M. Delbressine, M. Monincx, T. Hoveling, N. Meijer, C. Bangaru, J. Sterk, D. A. A. van der Woude, S. G. Oei, M. B. van der Hout-van der Jagt

**Affiliations:** ^1^ Department of Industrial Design, Eindhoven University of Technology, Eindhoven, Netherlands; ^2^ Department of Obstetrics & Gynecology, Máxima Medisch Centrum, Veldhoven, Netherlands; ^3^ Department of Obstetrics & Gynecology, Amphia Hospital, Breda, Netherlands; ^4^ Department of Electrical Engineering, Eindhoven University of Technology, Eindhoven, Netherlands; ^5^ Department of Biomedical Engineering, Eindhoven University of Technology, Eindhoven, Netherlands

**Keywords:** perinatal life support, extra-uterine life support, medical prototype, transfer devices, medical simulation, medical design, participatory design

## Abstract

**Introduction:**

Extra-uterine life support technology could provide a more physiologic alternative for the treatment of extremely premature infants, as it allows further fetal growth and development ex utero. Animal studies have been carried out which involved placing fetuses in a liquid-filled incubator, with oxygen supplied through an oxygenator connected to the umbilical vessels. Hence, by delaying lung exposure to air, further lung development and maturation can take place. This medical intervention requires adjustments to current obstetric procedures to maintain liquid-filled lungs through a so-called transfer procedure.

**Methods:**

Our objective was to develop obstetric device prototypes that allow clinicians to simulate this birth procedure to safely transfer the infant from the mother's uterus to an extra-uterine life support system. To facilitate a user-centered design, implementation of medical simulation during early phase design of the prototype development was used. First, the requirements for the procedure and devices were established, by reviewing the literature and through interviewing direct stakeholders. The initial transfer device prototypes were tested on maternal and fetal manikins in participatory simulations with clinicians.

**Results & discussion:**

Through analysis of recordings of the simulations, the prototypes were evaluated on effectiveness, safety and usability with latent conditions being identified and improved. This medical simulation-based design process resulted in the development of a set of surgical prototypes and allowed for knowledge building on obstetric care in an extra-uterine life support context.

## 1 Introduction

Extremely preterm birth, constituting 5% of preterm births globally, is the primary cause for high morbidity and mortality in newborns (1), and long-term morbidity later in life (2, 3). Organ immaturity makes the transition from fetal to neonatal life particularly difficult for extremely premature infants (<28 weeks). Neurodevelopmental disorders, respiratory diseases, sensory impairments, cerebral palsy, bowel syndromes and cardiovascular complications belong to the most prominent health issues (2). Despite the improvements made in neonatal intensive care units (NICU), approximately 60% of infants surviving will endure life-long complications (1, 2). Current neonatal intensive care for these newborns can include mechanical ventilation, which may exceed the developmental maturity of certain organs (4–6).

To advance the care for these infants, several research groups are investigating the possibility to mimic the uterine environment more accurately, allowing the infant to maintain fetal physiology to extend organ maturation (7, 8). This involves providing the infant oxygenation and nutrition through the umbilical vessels via an Artificial Placenta (AP) whilst the infant, or in different words: perinate (9), is placed in a fluid-filled incubator, an Artificial Womb (AW) system (APAW). The development and use of this extra-uterine life support technology has been achieved in animal studies, mostly lamb and piglet models (10). It proved to be successful in several aspects, including maintenance of circulation for up to 4 weeks, and demonstrated normal lung maturation (7).

Despite the promising research in animal studies, certain challenges remain to be solved for this technology to be translated to humans. In this study we take a design-perspective and particularly focus on the obstetric procedure, which will require adjustments to human patient care standards (9, 11).

A milestone that needs to be covered in APAW treatment is the prevention of fetal to neonatal transition, which normally occurs at birth (12). This transition is thought to be triggered by a multitude of factors, including change in skin temperature, sensory stimulation, chemoreceptor stimulation, placental hormones, cord occlusion, liquid clearance of the lungs, with the initiation of breathing thought to be a catalyst (12–18). Adjustment of the birth process, a transfer procedure, should ensure that the infant is transferred from the native uterus to the APAW while maintaining fetal physiology (9). The possible adverse outcomes associated with premature lung aeration, such as oxygen toxicity or interruption of fetal circulation when linked to an artificial placenta, indicate the necessity to refrain from lung aeration during the transfer procedure. While pharmacological suppression of breathing has proven effective in animal studies, other methods to suppress respiration also need to be considered. Next to preventing lung aeration, measures should be in place to maintain infant skin temperature when delivered from the birth canal or the uterus via cesarean section (CS). Thirdly, occlusion of the umbilical cord (UC) must be prevented as oxygenation will continue to be provided via the placenta until the circulation can be bypassed onto an artificial placenta. The perinate should be connected to the oxygenator before separation of the placenta occurs and the flow of oxygen- and nutrient-rich blood of the mother is greatly diminished. Lastly, external factors such as mechanical trauma, infection risk, and strong light, and sound directed to the perinate should also be minimized.

Animal studies of APAW technology have successfully placed animals, via (adapted) cesarean section (CS), on extra-corporeal support (7, 19–21). As researchers in the field noted, participation in clinical trials of this technology would determine the mode of delivery for mothers who could otherwise have given vaginal births (9, 22). Therefore, from a maternal perspective, transfer via vaginal birth (VB) should also be investigated, as VB should pose no additional risk to the mother, unlike a CS (22). Exposure to vaginal flora may contribute to developing the newborn immune system (23–25). Therefore, contact with this microbiota, whilst preventing risk to early-onset-sepsis, could be further explored (26).

Transfer by VB brings additional challenges, such as increased risks for infection, prolonged labor and a generally less-controlled environment compared to CS. Maintaining the sterility of the environment is crucial, especially since chorioamnionitis is responsible for triggering roughly half of preterm births (27). Nevertheless, to improve success rates and because roughly half of preterm births are delivered with CS (28), a transfer via both CS and VB should both be investigated (9). The development of a human-tailored obstetric transfer procedure requires substantial investment of time and resources and would benefit from involvement of a range of stakeholders. Also, research ethics for testing such a high-risk procedure in human patients is still in development (29).

n this study we created a simulated transfer procedure for both VB and CS with obstetric device prototypes. We employed a simulation-based design method to investigate how to facilitate the transfer, lower the chances of unwanted neonatal transition, complementing research performed in animals (11). The instruments used in the medical simulations explicitly constitute prototypes rather than certified medical devices. The prototypes and procedural tasks should ensure prevention of neonatal transition, thereby shielding the perinate from external stimulants, such as aeration of the lungs, whilst also attenuating stimulation of light, sound, temperature, and handling. In this study, we present the design process and performance assessment of these instruments within the framework of simulation. We provide a description of the simulation-based development process, concepts, fabrication, and risk evaluation.

## 2 Materials & methods

### 2.1 Design process

The research went through multiple phases, with each phase requiring several iterations (see Supplementary Figure S1). First, from a literature study and expert interviews requirements were identified. The most promising design concepts were selected by expert consulting. Concepts were evaluated on safety, hygiene, comfort, user experience and transfer success. In this study a simulation-based development method was taken (11), therefore fabrication was followed by a first round of user tests with fetal and maternal manikins. A Failure Mode and Effects Analysis (FMEA) was performed to reveal risks and mitigation strategies (30). This FMEA analysis has multiple steps: a Hierarchical Task Analysis (see Supplementary Table S2) gave an overview of all tasks, and a Fault Tree Analysis (see Supplementary Table S3) revealed possible dangers like injury and impaired efficiency and ergonomics (31). Hazards were ranked based on severity and probability using a risk assessment matrix. Mitigations for the hazards were drawn up and procedural and design adjustments were made accordingly, after which final liquid-based simulations were performed (6).

### 2.2 Fetal and maternal manikins

MRI data of a fetus of 24 weeks gestational age were used to develop a mold to cast a silicone manikin (Ecoflex 00-30, Smooth-on, USA). For the maternal manikin, a PROMPT Flex Birthing Simulator (Limbs & Things, United Kingdom) was used. The PROMPT simulator was 3D scanned (Eva Scanner, Artec 3D, Luxembourg) to enable subsequent 3D modelling and 3D printing of a custom watertight abdominal insert (PLA filament, Ultimaker, the Netherlands). This insert, including tissue layers and a uterus, was developed to allow for increased simulation realism and validation of the prototypes. The skin layer, with a premade skin incision, consisted of a top layer of silicone Ecoflex 00-30 (Smooth-on, Macungie, USA), glued on top of 2 cm thick polyether foam. A bag, functioning as amniotic sac was placed in the uterus and could be interchanged and varied in size based on gestational age of the fetal manikin.

### 2.3 Expert interviews and simulations

Different study stages included semi-structured interviews and simulations. Simulations increased in complexity and number of participants throughout the process, starting with dry simulations (no “amniotic fluid”) in the beginning, to liquid-based simulations at the final stages of the design process. Participants were selected for clinical expertise or preterm delivery/care experience. Animations or concept drawings of transfer procedures and equipment were displayed in interviews. Participants tested low- to high-fidelity prototypes on manikins in simulations.

### 2.4 Fabrication of transfer device prototypes

Fabrication of transfer device prototypes took an iterative approach. The set of instruments for the CS include: two wound retractors, a retractor connector and a transferbag with integrated gloves. An inflatable wound retractor and transferbag with integrated gloves are included for VB. The transfer station adjusts for delivery type, keeps the transferbag near the placenta, with attachment for the oxygenator. The fabrication of all these prototypes is described below. A list of anatomical and (bio)mechanical parameters on which design decisions were based can be found in Supplementary Table S1.

#### 2.4.1 Transferbag

First a scaled prototype made of silicone was developed through casting in a 3-part mold. This proved overly heavy, bulky, and complex to fabricate the integrated gloves necessary for the obstetrician's dexterity in delivering the perinate from the uterus to the transfer bag. Therefore, a 1:1 prototype of the transferbag was made from 0.1 mm thick thermoplastic polyurethane (TPU) film and sealed via a combination of heat laminating using a laser engraver (Trotec Speedy 300, Austria) and ultrasonic plastic welding (SSU-015R, Sew Systems, UK). The rigid ring attached to the transferbag was printed on an Objet Connex 350 (Stratasys, Israel) using a mixture of Vero White and Tango Black material (Stratasys, Israel), a photosensitive resin that creates plastics and rubber-like materials. The transferbag ring attaches to the retractor connector, with a rubber labyrinth seal and additional O-ring 3D printed as an assembly to the rigid ring, to ensure a liquid-tight seal (Tango Black material, Stratasys, Israel). The basic design of the transferbag with integrated gloves, air valve and liquid supply/drain inlets is similar for VB and CS.

#### 2.4.2 Retractors

##### 2.4.2.1 C-section

**Figure 1:**
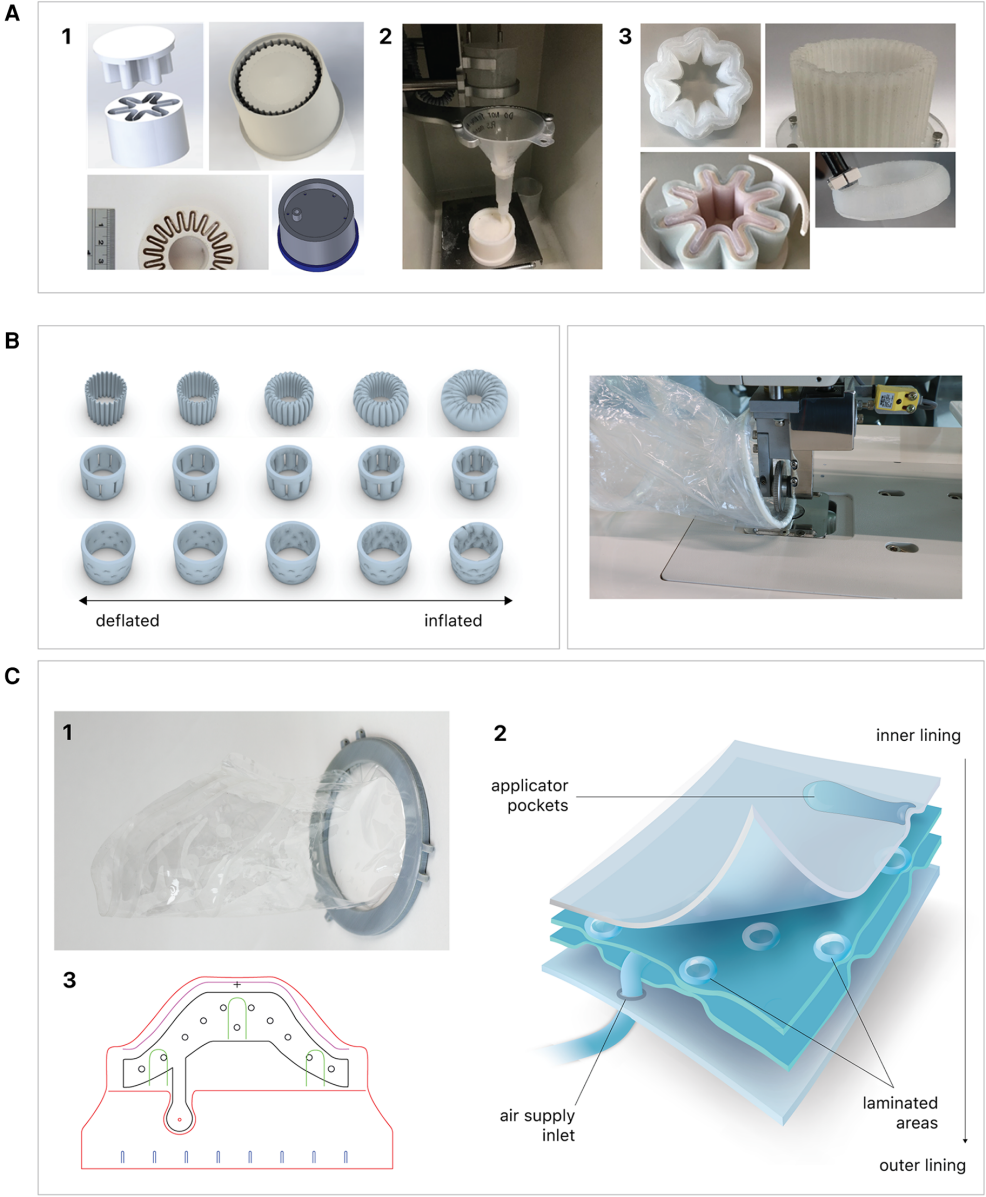
Fabrication of the inflatable retractor. **(A)** (1) Multi-part molding and (2, 3) silicone casted retractors, **(B)** digital simulation of inflation of variations in areas of lamination and resulting air chamber geometries, **(C)** fabrication of TPU lamination (plastic welding pictured) and **(D)** (1, 2) the final multi-layered TPU retractor prototype and (3) 2D lamination pattern.

**Figure 2:**
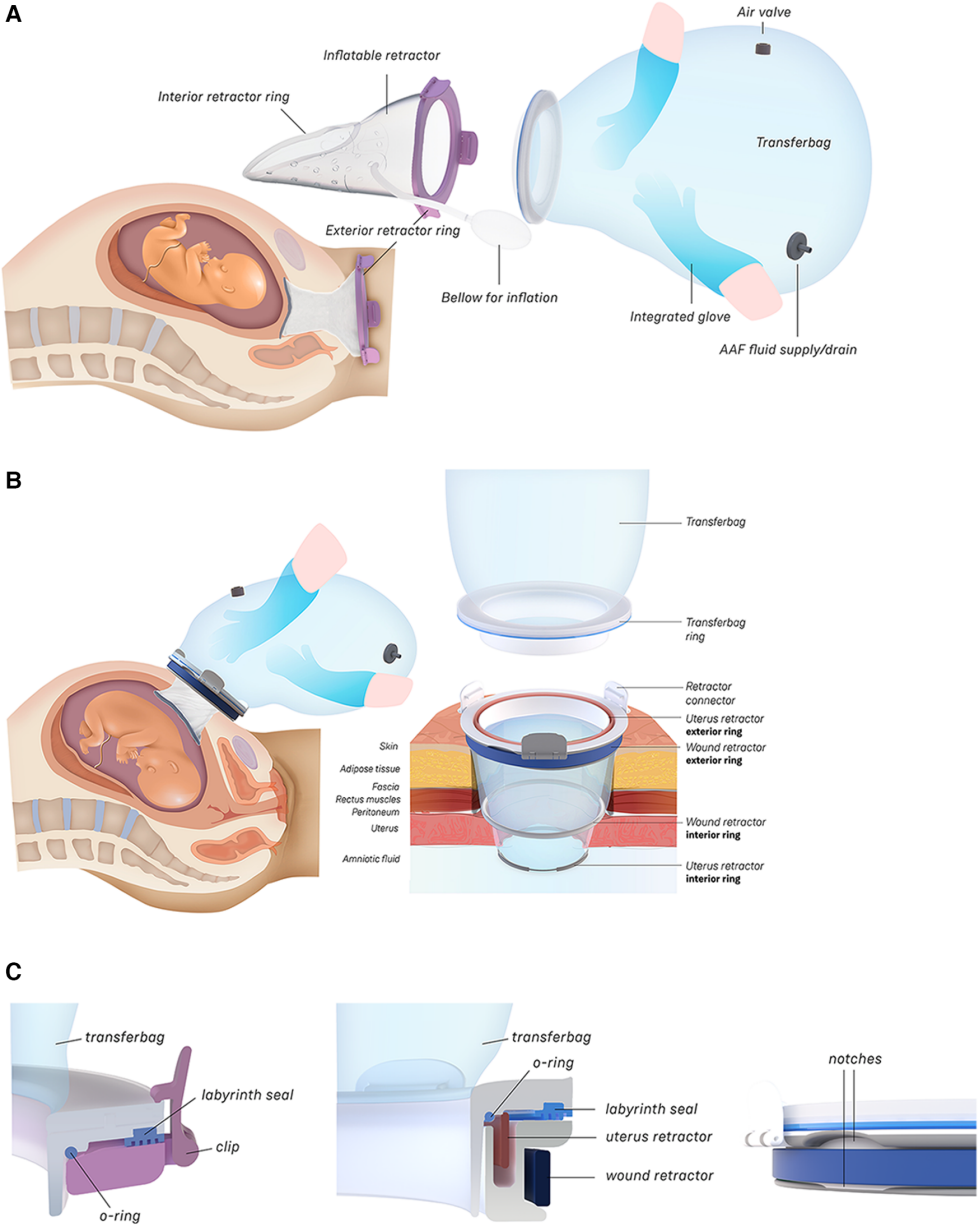
**(A)** Design of the transfer device prototype for vaginal birth. **(B)** Design of transfer device prototype for cesarean birth. **(C)** Design details of the VB device (left) and the CS device (center, right).

**Figure 3:**
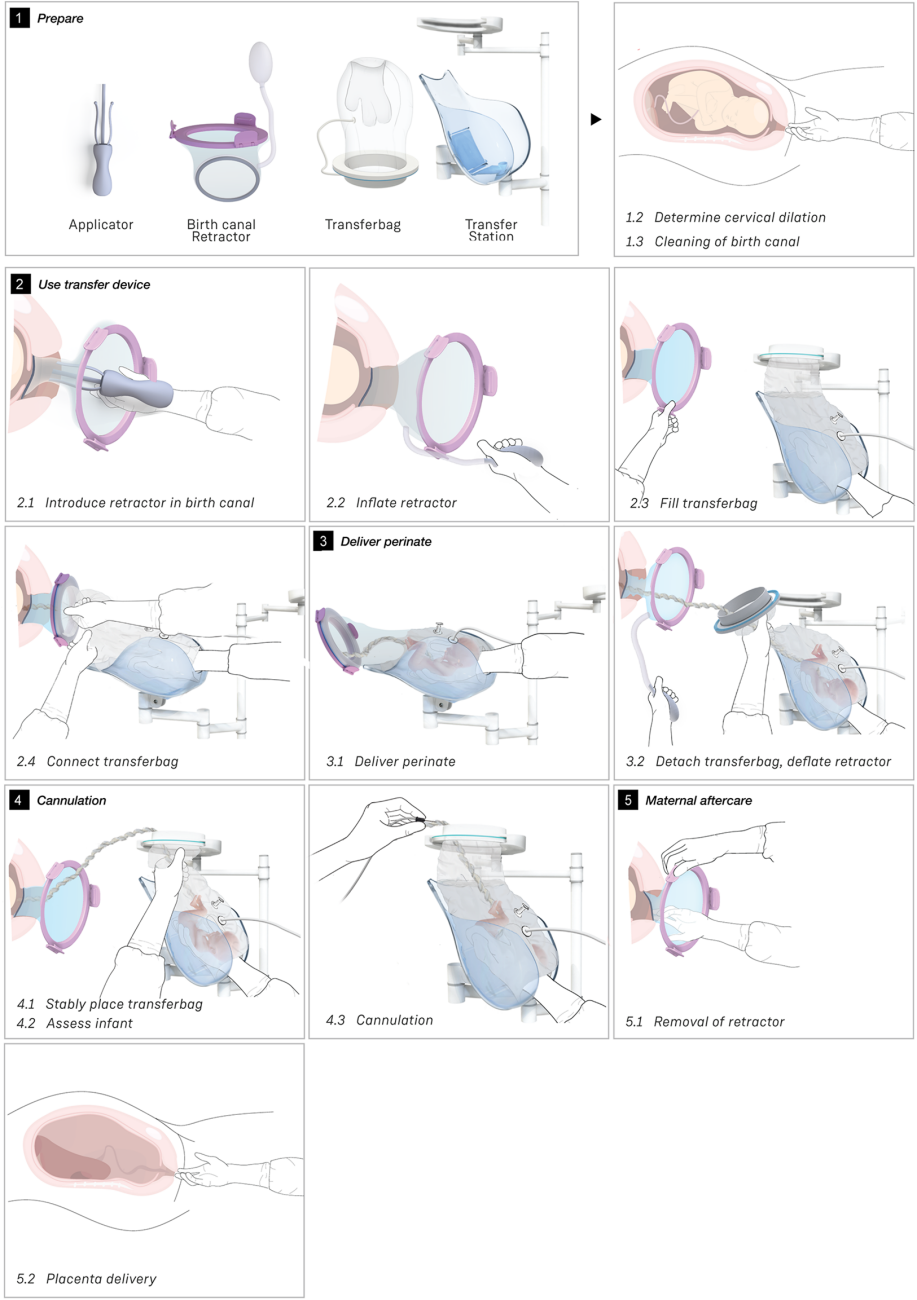
Sequences of the simulated transfer procedure (VB).

For the CS transfer device prototype, a commercially available protractor-retractor (SurgiSleeve, Medtronic, USA) was used. A uterus retractor was created from 0.05 mm thick PE film that was ultrasonic welded (SSU-015R, Sew Systems, UK) and attached to two flexible 3D printed TPU rings (TPU, Ultimaker, The Netherlands) (see Figures 1–3). The ring inserted within the uterus has a diameter of 80 mm for the infant to smoothly pass through. Infants of 24-week GA have an average head circumference of 222 mm (32), yielding an approximate estimate of 70 mm diameter. The ring itself has a thickness of 4 mm with thinner regions at two places to fold when placed into the uterus (see Figure 2B). The exterior ring has a diameter of 164 mm and fits within a notch of the retractor connector. The retractor connector was designed to enable attachment of the exterior ring of the Surgisleeve and the exterior ring of the uterus retractor, and to attach/detach the ring of the transferbag using three clips. The retractor connector and clips were 3D printed using a mixture of Vero White and Tango Black material (Stratasys, Israel) on an Objet Connex 350 (Stratasys, Israel).

##### 2.4.2.2 Vaginal birth

Currently available devices for use in instrumental delivery include e.g., forceps, vacuum, wound retractors, and the Odon device (33, 34). Another recent experimental design is that of a cervix dilator to better enable delivery during breech delivery (35). A retractor was taken as a starting point in the design process. Multiple methods, molds, geometries, and materials were employed to construct an inflatable retractor for VB. The geometry of the birth canal is shaped as a cone (36). The average (non-pregnant) cervix has an ellipsoid shape with a diameter of 25 by 30 mm. In theory, the average cervix, vaginal canal, and vulva enlarge to 100 mm diameter in term delivery (37, 38), for a 38-week GA child with a head circumference of 342 mm (32). For an extreme preterm birth, we estimated that 100 mm outer diameter and 80 mm inner diameter (251 mm circumference) of the inflated retractor would be sufficient for the present simulation study. Initial prototypes used silicone casting in multi-part molds (see Figure 1). Multiple inflated chambers instead of a single chamber proved the best way to prevent undesirable radial expansion of the inner wall (see Figure 1C). Main fabrication issues were related to the entrapment of air bubbles in the thin channels of the mold, which could partly be solved using a vacuum casting machine (340 Multiplex, Schuchl, Germany). It proved more successful to create prototypes by laminating several films of TPU compared to casting silicone inflatables. Retractor sleeves with two TPU films (an envelope) and a variety of inner air chamber designs were created and tested on their expansion effect. Simulation software was used during the form finding process, to visualize the pneumatic structures' behavior under air pressure (see Figures 1, 2). First, 3D designs of envelopes were created and then using a modified Grasshopper script and Kangaroo Physics plugin, their behavior was simulated under the load of inflation (Grasshopper, McNeel Rhinoceros) (39).

**Figure 4:**
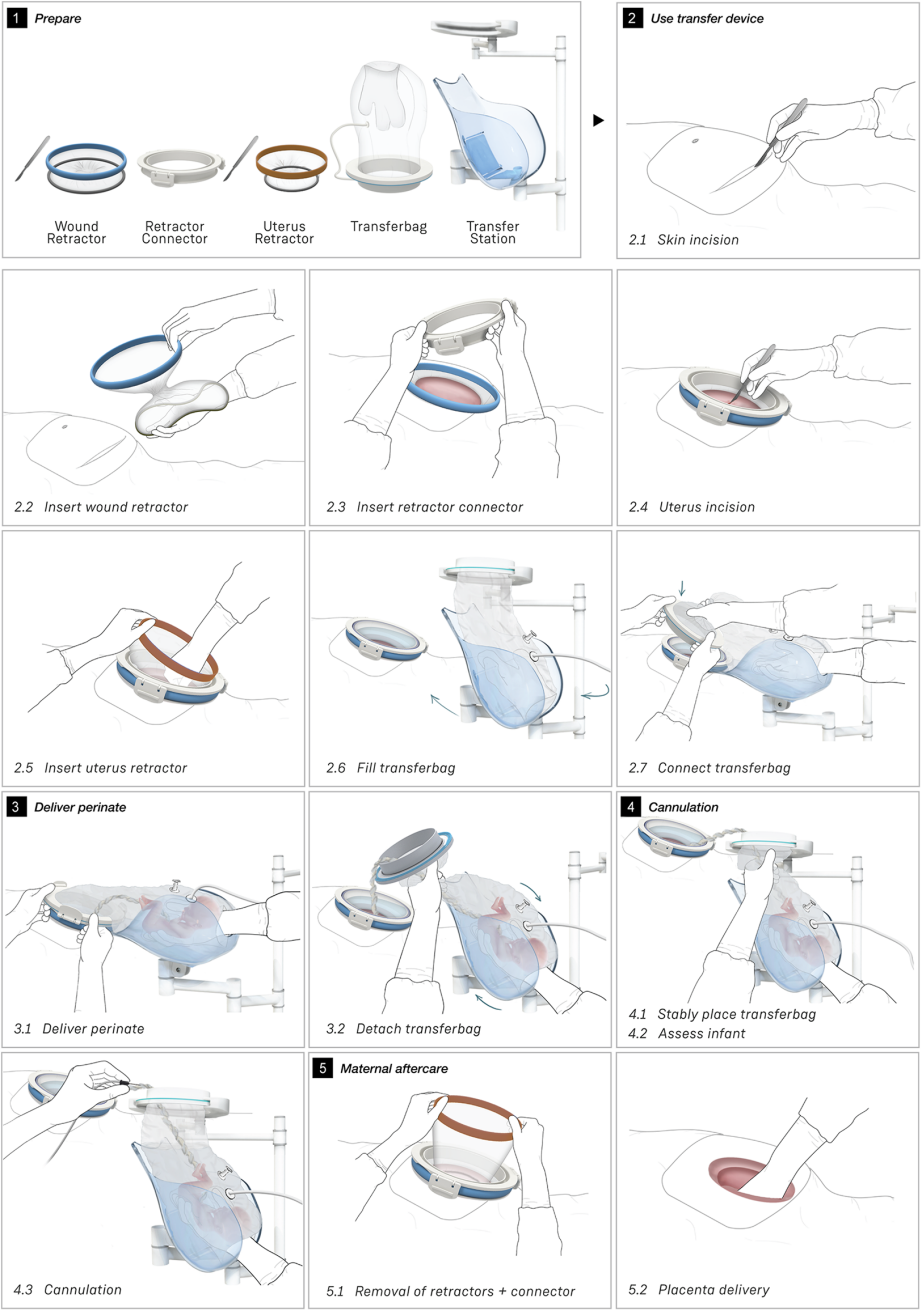
Sequences of the simulated transfer procedure (CS).

The design that contains circular laminated regions appeared to be the most promising. To fabricate the sleeve, a 2D vector file of the 3D design was made, which could be cut, and pattern-engraved on a laser engraver (Trotec Speedy 300, Austria) (see Figures 1–4). To create a cylinder of the flat sheet, the TPU film was formed in a 3D shape and sealed using an ultrasonic welder [SSU-015(R), Sew Systems, United Kingdom]. The exterior part of the retractor was attached to a rigid ring, connectable to the transferbag. A bellow was connected to the opening to allow for inflation of the air chamber. Due to the curved shape of the birth canal towards the cervix, the sleeve was designed with a diagonally cropped top to avoid unnecessary piling of the material in front of the cervix. The circular shape of the bonded areas ensures that the pressure applied to the lamination is evenly distributed, when compared to the 90° corners in the case of columns.

In another study, this approach of a hexagonal lattice pattern has already proven useful for the fabrication of inflatable stents (40). The continuous network created through the pattern of offset laminated circles is more efficient than in the case of isolated individual chambers, both in terms of inflation as well as ease of fabrication. For this proof-of-concept, attention was given to using a pattern that exerts sufficient radial force upon inflation to be kept in place during delivery. To minimize the chance of buckling, an alternating pattern is used.

Taking inspiration from the design of the Odon Device (33), an applicator with three flexible arms was constructed (TPU, Ultimaker, The Netherlands) for accurate placement of the inflatable retractor in the birth canal. For the same reason, a fixation ring (TPU, Ultimaker, The Netherlands) has been embedded in one end of the retractor sleeve.

#### 2.4.3 Transfer station

**Figure 5:**
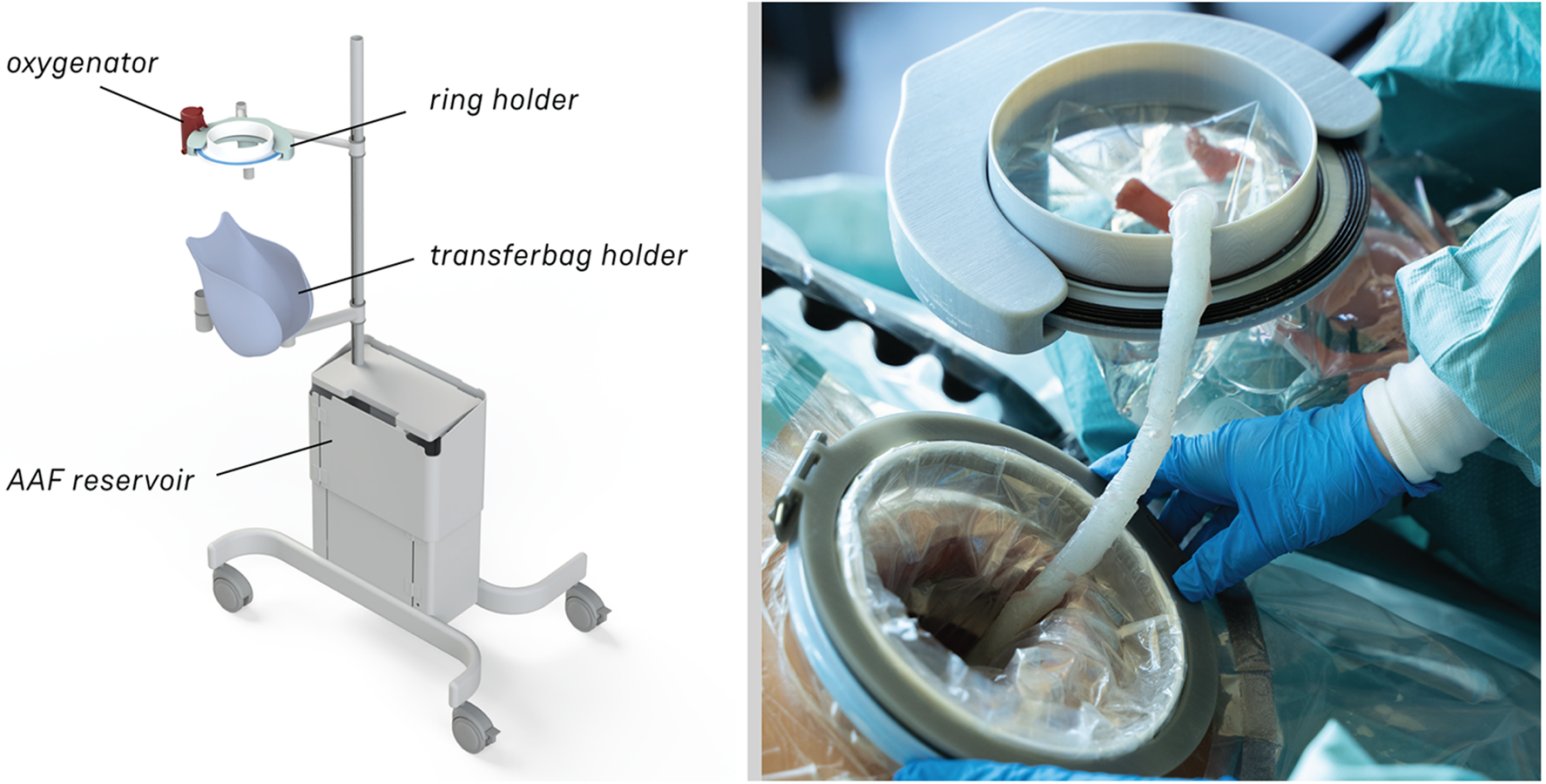
**(A)** Design of the transfer station that allows proximity to the maternal site with transferbag holder, oxygenator placement. **(B)** The transfer station in practice during a simulation.

The transfer station consists of 5 different elements: a base with attachment pole, a platform for liquid-filled-lung intubation, a holder for the transferbag, a ring holder and a reservoir (see Figure 5). The metal base with wheels was disassembled from an existing incubator and re-assembled with a pole to which metal arms with joints were mounted, which can be rotated and tilted. The arms are of sufficient length (650 mm) to allow for close placement to the mother. The platform for the liquid-filled-lung intubation was 3D printed in Nylon (Ultimaker, the Netherlands) and fixed on one arm using a custom-designed slide mechanism 3D printed in acrylonitrile butadiene styrene (Fortus 250mc, Stratasys, Israel). The holder for the transferbag was also 3D printed in Nylon (Ultimaker, the Netherlands). The ring holder, 3D printed in an ABS-like resin (Verowhite, Stratasys, Israel) on an Objet Connex 350 (Stratasys, Israel), was designed to allow placement of the transferbag at an angle to prevent the transferbag from sliding out.

### 2.5 Failure mode and effect analysis

The procedures and handling of the transfer device prototypes contain many steps, different instruments, and require teamwork. By performing risk assessments early in the development process, possible flaws and their associated hazards can be identified, making it much easier to minimize error-prone aspects of the design (41, 42). In high-stress environments, this novel procedure potentially presents future operators with a high (mental) workload and associated safety risks, highlighting the critical importance of device usability for ensuring safe and efficient procedures (43). A Failure Modes and Effect Analysis (FMEA) provides a systematic framework for the analysis of the potential failures, their causes, and their effects on the entire procedure: (1) task identification (2) hazard identification, (3) risk assessment, and (4) risk mitigation in case of unacceptable risk levels (42). We made use of the ISO 14971:2019 standard for the application of risk management.^1^

#### 2.5.1 Hierarchical task analysis

A Hierarchical Task Analysis (HTA) broke down the procedure into a hierarchy of tasks and subtasks for each user (see Supplementary Table S2) (44, 45). User task allocations were iteratively refined through a process of expert reviews and dry simulations.

#### 2.5.2 Hazard identification

Potential failures were identified using a Fault Tree Analysis (FTA) (see Supplementary Table S3) (46, 47). A Fault Tree Analysis begins with a potential failure and reverse-engineers its cause. This is helpful for novel interventions without historical data to review. Of obstetric errors, 75% are caused by a lack of teamwork and communication skills (48). For this analysis we started with the division of potential human errors (including team communication) and device-related errors. Simulations (phase 3) identified potential device-related errors caused by design, manufacture, or inappropriate usage, and human errors caused by wrong action or inaction that might threaten patient safety (49). The interaction qualities of the device prototypes and the procedure could be analyzed using a role-playing method involving both researchers and participants (44).

#### 2.5.3 Risk assessment

Part of the risk management is the risk analysis which investigates how much risk is associated with the identified hazards. Using a risk assessment matrix, judgements were made on the tolerability of the risks (see Supplementary Table S4). Due to the unprecedented nature of this procedure, and since no historical data is present, expert judgment was chosen (30). The matrix was based on a typical risk matrix (ISO14971) (30) that breaks down the severity and probability axes into discrete scales; the severity in major (4), serious (3), minor (2), incidental (1), and probability in frequent (4), occasional (3), seldom (2), unlikely (1). Using the FMEA framework, the FTA and risk assessment were combined in a worksheet (30) (see Supplementary Table S4). There is no set standard for the worksheet, since each analysis asks for different components. The general features are description of unit, description of failure, effects of failure and possible mitigations (30). For this analysis we intended to reduce high-severity risks, therefore we gave severe events greater weight despite their lower chance by altering the risk matrix' major severity weight from 4 to 12 (see Supplementary Tables S3A and S3B).

### 2.6 Evaluation and validation through simulation

The first set of simulations were dry simulations, hence performed without filling the intra-uterine amnion bag and transferbag with water. The simulation was conducted with a total of five participants (P29–P35), including three perinatologists, a technical physician, and a medical engineer also educated as a midwife. Sessions took place at the Dept. of Industrial Design (Eindhoven University of Technology) and at a medical simulation center (Medsim, Eindhoven, the Netherlands). The sessions involved the use of fetal and maternal manikins. Participants were instructed on procedural tasks, tool handling and their place within the entire clinical team. Only tasks related to non-pharmacological treatments were included. Throughout the protocol, discussions were stimulated, and idea generation was facilitated using the prototypes. Illustrations, animations, or live instructions showed participants procedural tasks, device handlings, and operational hierarchy. Task cards with checklists helped participants complete tasks in correct sequence throughout the simulation (adapted from Figures 3, 4). Liquid-based simulations sessions were held with seven participants (P43–P49). Also, within these simulations, the simulation of pharmacological treatments was omitted. Liquid-based transfer simulations included in this study were only for CS, not (yet) for VB which included only dry simulation.

During the first set of simulations, photos of prototype interaction and procedural tasks were taken of the participants. Minutes were drawn up after each session. Of the liquid-based simulations transcripts were made from the (audio/video) recordings, in certain cases recording was not possible and minutes were made directly after the session. The knowledge gained through interviews and simulations was continuously compared to previously collected information. Through data and source triangulation internal validity was assured. Data triangulation was assured using a variety of methods such as simulation, individual and group interviews. Source triangulation was assured by including specialists from different disciplines and sources of experience.

Testing the approach of filling only the lungs instead of submerging the entire perinate's body with liquid, is beyond the scope of this paper.

## 3 Results

### 3.1 Simulation procedure criteria

For the present study we focused on infants of 24 weeks gestational age, in line with current Dutch guidelines on care at the edge of perinatal viability. Past preclinical studies have shown successful extracorporeal support up to 4 weeks, and therefore perinates between 24- and 28-weeks of gestational age have been the target group for this study, in relation to development of the Perinatal Life Support system (9). Expert input led to various assumptions to guide procedure development within specified boundaries for the first patient group to be addressed. Twin pregnancy is not included, both mother and fetus are healthy and stable, the fetus is in cephalic presentation, the placenta allows VB and CS, the umbilical cord is not entangled and the perinate is cannulated while ex-utero. Future studies should include a wider range of scenarios and should include cannulation while the fetus is still in-utero, similar to EXIT-to-ECMO procedures to account for uterus involution and shearing of the placenta. The obstetric procedure for perinate placement in the APAW system requires an adaptation from current preterm care. It should prevent transition to neonatal physiology, facilitate rapid umbilical vascular cannulation to the extracorporeal circuit and shield the perinate from environmental factors such as temperature shifts, mechanical trauma, sound, and light (9).

What factors initiate neonatal transition is not entirely understood. Although the suppression of the breathing reflex could be maintained by administering certain drugs, the strategy behind this study was to envision a method to prevent transition through non-pharmacological means. In both modes of delivery these interventions can be ensured by creating (1) an air and watertight artificial passageway, either in the birth canal or via the abdominal incision to access the uterus. This passageway should shield the perinate from air, but also keep the environment sterile by preventing exposure to e.g., maternal pathogens (Group B strep), which should be of extra concern for transfer by VB. The environment in which the perinate is captured, the transferbag, should (2) allow for the supply of warmed artificial amniotic fluid (AAF). Lastly, the procedure should (3) allow the umbilical cord to be cannulated in proximity to the placenta due to its limited length, which is 40 cm on average at 24 weeks of GA (45).

Based on the expert interviews and the subsequent simulations, a list of general procedural and design requirements was drawn up, see Table 1. For a comprehensive list see Supplementary Table S1.

**Table 1:** Requirements.

Category	#	Requirement
Simulation protocol	1	Procedure is integrated within current obstetric workflow for premature birth. For CS this could include ex utero intrapartum treatment
Simulation protocol	2	Mental workload required for the procedure is not significantly increased with respect to other clinical procedures
Simulation protocol	3	Procedure can be stopped (and diverted to conventional care) or temporarily interrupted in case of: umbilical cord compression, initiation of respiration, other cardiovascular complications, or signs of pathology
Cannulation	4	The cannulation should occur in proximity of the mother, as umbilical cord length at 24 weeks GA is roughly 40 cm (45)
Cannulation	5	Umbilical vessels are connected to machinery of the APAW system through cannulation (7)
Cannulation	6	Cannulation is performed when perinate is ex-utero, as in-utero cannulation could potentially lead to decannulation events upon delivery
Cannulation	7	Cannulation is ideally performed within a few minutes after delivery to prevent asphyxia due to vasoconstriction of the umbilical cord (50)
Simulation protocol	8	The procedure should ideally be available for both VB and CS
Simulation protocol	9	In standard CS, time from first incision to delivery takes about 5 min (51)*.* The transfer from the native womb to transferbag to APAW system should be well-timed
Simulation protocol	10	Suspected fetal distress before starting the procedure is a contraindication. The position and station of head within birth canal should be determined
Transition prevention	11	Perinate's lungs should be shielded from air and maintain fetal physiological state. Environmental factors should be attenuated (light, sound, mechanical trauma or pressure)
Trauma prevention	12	Avoid physical damage to mother's tissue. Friction between device and tissues should be minimized during the procedure to prevent skin damage (e.g., perineal rupture)
Trauma prevention	13	Performed mechanical forces on the baby should be limited to avoid birth trauma (52). The head of the premature perinate is especially fragile, with a high risk of an intraventricular hemorrhage
Well-being	14	Provide adequate support care to parents
Hygiene	15	Direct contact of the fetus with the birth canal and/or vagina should be prevented to protect against pathogens such as GBS, E. coli and pseudomonas
Hygiene	16	Contains no small ridges, holes, sharp edges, or other potential areas for bacterial accumulation (53)
Hygiene	17	The procedure can be made sterile (as feasible) to mitigate the risk of infections
Design	18	The environment is translucent for medical staff to observe the perinate and can also be made light and sound blocking for the perinate
Design	19	The material of the product is medical grade, non-toxic, sterilizable, soft and smooth, and well resistant to fluids, lights, and a large range of temperatures
Design	20	The product contains connections for the supply/drain of the AAF
Design	21	The product should at least be filled with an AAF volume of 600–800 ml (24th and 28th week of gestation) (54)
Design	22	The temperature of the AAF in the bag can be controlled
Design	23	The device has handles or a controllable surface to avoid slipping and to improve steerability
Design	24	Perinate can be moved around within the transferbag. This might become necessary in case the perinate is wrongly positioned
Design	25	The procedure should avoid tangling or other issues that could lead to the occlusion of the umbilical cord
Design	26	During a CS transfer, blood accumulation from the incision sites entering the liquid environment should be avoided
Design	27	Excessive leakage of AAF into the maternal abdominal cavity and wound edges should be avoided

### 3.2 VB transfer device design

Wound protector-retractors provide circular retraction by separating the edges of the incision, hence moving tissue aside to improve access. In general, retractors exist of two flexible rings that are connected by a cylindrical plastic film, thereby forming a tunnel that can be placed in bodily cavities. Using the wound retractors, a watertight artificial passageway can be created. The outer end of the retractor forms an interface onto which other instruments can be attached, such as a liquid-filled transferbag. After delivery, by removing the transferbag from the birth canal, the umbilical cord is exposed and can be cannulated while placing the transferbag on a stable platform in proximity to the mother (see Figure 3).

In this study an inflatable retractor was created to form an airtight passage from the uterus to the transferbag. Accordingly, the retractor was designed to secure against the birth canal whilst reducing maternal stress and perineal damage. Pressure closure and thereby automatic adjustment and comfort to surrounding tissue and anatomic structures favored inflatable constructions. Inflatable constructions were preferred for their pressure closure mechanism, enabling automatic adjustment to and comfort in the surrounding tissue and anatomical structures. In addition, they minimize the chance of trauma by lacking sharp or rigid elements.

One of the first iterations for the retractor design allowed the retractor and transferbag to be connected and attached to a handle (see Supplementary Figure S2). The handle hosted the air and fluid supply/drain tubing. Upon entry in the birth canal, the retractor could be inflated, and the perinate could then slide into the transferbag. Having all components (retractor, transferbag, supply/drain, user interface) in a single device made it easier to operate, but it also had several drawbacks related to sterility and accessibility to measure cervical dilation. In the final design (Figure 2), integrated gloves in the transferbag replace the device handle to help direct the perinate into the bag. Separating the transferbag and retractor permits the retractor to be placed in the birth canal and upon sufficient cervical dilation, the retractor can be inflated and the transferbag attached. Additionally, sterility can be better maintained since the transferbag has less contact with the birth canal.

In the final prototype, manufacturing of the inflatable retractor is based on directed lamination of two plastic films, containing air pockets and bonded areas. The inflatable retractor's asymmetrical sleeve conforms to the birth canal's shape, therefore this emphasizes the necessity for proper insertion by the user into the manikin's birth canal.

One way to reduce degrees of freedom during insertion was by pre-assembling the retractor with an applicator. Three flexible arms are placed in the inner pockets of the sleeve which can be pulled out easily afterwards. The handle secures that the sleeve can only be inserted in the right way. The final prototype has an additional fixation ring that is embedded in one end of the retractor that, while pre-folded upon entry in the birth canal, will unfold around the cervix to allow passage of the perinate (see Figure 2A). During insertion, the retractor is pushed up towards the cervix, by the clinician's index and middle finger. The other end of the retractor, outside the vulva, is connected to a rigid ring onto which the transferbag can be attached. This transferbag is again made of TPU with two integrated gloves, an inlet for AAF supply and a valve to release trapped air.

The length of the umbilical leaves limited space to perform the cannulation if the perinate is already delivered in the transferbag. Hence, we designed a transfer station comprising an adjustable platform for supporting the transferbag, an integrated reservoir for heated AAF supply and a holder for the oxygenator during perinatal umbilical cord cannulation.

Figure 3 shows the sequential steps of the VB transfer procedure that were performed during the simulation. In case of anticipated preterm birth, the position of the fetus and placenta is assessed by ultrasound, and routine fetal and maternal monitoring is used. The perinatologist determines the required cervical dilation based on gestational age and perinate head size. After cleaning the perineum and vulva, the interior ring of the inflatable retractor is introduced in the birth canal, while the exterior ring remains external. The transferbag is pre-filled and placed on the transfer station while one perinatologist places one hand in the integrated glove. After membrane rupture, the transferbag is connected to the retractor. An air valve in the transferbag releases trapped air and sufficient AAF is supplied. Uterine contractions, maternal pushing, and guidance by the clinician allow the perinate to slide into the birth canal and subsequently into the transferbag. The transferbag is then detached from the retractor and placed in the holder to prevent fluid leaking and to allow visual examination of the perinate. To create an extracorporeal circuit with the AP, the umbilical vein and arteries must be readily cannulated. The perinate is transferred to the APAW system where monitoring, oxygenation, and nourishment of the perinate is taken over. The perinatologist guides placenta delivery and sutures perineal tears if required.

### 3.3 CS transfer device design

To broaden the applicability of this treatment, and because half of premature infants are born via CS (2, 8), we investigated the possibility of a transfer via CS. Making use of the same concept, a passageway is created to which a liquid-filled transferbag can be attached. Following skin incision, an existing wound retractor SurgiSleeve (Medtronic, USA) can be used to obtain a clear operating field. Subsequently, the retractor connector can be placed onto the outer facing ring of the retractor. The inner ring is located tight against the peritoneal layer. A second wound retractor is placed after the uterine incision is performed. This uterus wound retractor has the shape of a truncated cone, with the inner facing ring having a smaller diameter than the outer-facing ring. The outer facing ring can be placed in a notch in the retractor connector. A transferbag can connect to this set of tools, thereby creating a direct passageway from the uterus (see Figure 4).

Several design adaptations were made after the first rounds of simulations. To prevent the flow of AAF from the pre-filled transferbag into the abdominal cavity of the mother, the second wound retractor was added. With the additional retractor, potential inflow from blood from the incision wound edges into the uterine cavity is also prevented. This would otherwise mix with amniotic fluid and can then cause cloudiness and limit the view during the CS. Lastly, the wound retractors protect the incision wound edges against contamination. A second adaptation is related to the improved degree of freedom for the clinicians during the operation. During a standard CS, gynecologists need to make great use of their hand dexterity, however, the mounted transferbag onto the incision makes it harder to deliver the baby. Hence, gloves were integrated in the transferbag to reach for and guide the perinate, similar to the VB transferbag.

The retractor connector contains grooves for placement of the two outer-facing rings of the retractors (see Figure 3B). Rubber labyrinth and O-ring seals were placed at strategic places, on both the retractor connector and the transferbag to ensure watertightness (see Figure 2C). Three clips secure the transferbag onto the retractor connector, a reversible action when the perinate has been delivered. When the infant is delivered into the transferbag, the bag needs to be detached from the base by lifting it from the wound retractor, aided through the integration of notches (see Figure 2C).

The proposed transfer procedures require a specific sequence in tool usage. To enhance success and reduce cognitive load, this study underscored the importance of prioritizing usability and learnability. Design concepts like affordance (design elements that imply how to use an object) and signifiers (visual/auditory function indicators) provide the operator clues for usage and improve user experience (55, 56). We employed perceivable markers, such as labels and colors, to convey where and in what order an action should be performed. The design also includes affordances such as clips, gloves, but also geometric shapes in which the other elements can only be assembled in one manner.

Figure 4 shows the proposed sequential steps of the CS transfer procedure that were also performed in simulation. The mother is placed in 15 degrees left lateral tilt position (57) with body support components [e.g., an (inflatable) wedge]. Following the incision in the skin, the wound retractor and retractor connector are used. After the uterus incision, additional artificial amniotic fluid is supplied to the uterus, to keep the perinate's head submerged. The transferbag is filled with AAF and connected to the retractor connector. The mother is tilted to her left side and the perinate is guided into the transferbag. After placement into the holder of the transfer station, the umbilical vessels need to be rapidly cannulated to the oxygenator and the mother can be placed in a supine position. If needed, the transfer procedure can be halted at any moment during the procedure and a switch can be made immediately to standard preterm birth care.

### 3.4 CS and VB procedural characterization and failure mode effect analysis

Given the similarities between the designs, a joint FMEA and joint hazard mitigation were chosen. For each part it will be indicated whether it concerns CS, VB, or both.

#### 3.4.1 Task analysis

The main procedural tasks are distributed over two perinatologists who perform the delivery and operate the prototypes for both delivery modes (see Figures 3, 4 and Supplementary Tables S2A and S2B). Surgical assistant(s) are present to aid in handing device parts and provide support as needed. In addition to these healthcare professionals who have direct involvement in the device handling, a secondary group of potentially involved stakeholders may include an anesthesiologist, anesthesiology assistant, sonographer, neonatologist, neonatology resident, a nurse and a technical physician.

#### 3.4.2 Hazard identification

Potential hazards for both CS and VB were identified based on the dry simulations and expert consultation (P1–P35). The hazards include device-related risks as well as procedural risks (see Table 2).

**Table 2:** Overview of top potential hazards during the transfer procedure of both CS and VB based on our analysis.

Hazard	Description	Type	SEV	PROB	Criticality score
Transferbag is too heavy	The transferbag's volume could allow 7 liters. Holding this weight for a significant portion of the procedure might cause operator fatigue. In the worst scenario this could lead to dropping of the transferbag.	CS, VB	12	4	48
The perinate gets into contact with air	Sustained air exposure is unwanted. This could occur after uterine incision (CS), or after membrane rupture in vaginal birth, before the transferbag is attached, or if the trapped air is not released for other reasons (e.g., valve not operating). After connection of the transferbag to the retractor, additional AAF should be supplied and trapped air released, to account for the air present. Failure to do so may expose perinate to air.	CS, VB	12	3	36
Insufficient hand dexterity	Anatomical variations can lead to impaired dexterity and grip when the integrated gloves are too small/large. This may potentially cause delay, or even inability to deliver the baby (cancelled operation) or trauma if dropped.	CS, VB	12	3	36
The retractor ring is not pulled upwards / not inflated enough	To establish a tight transfer tool ensemble adequate retraction is needed to limit material buildup, enable incision site tamponade to decrease blood accumulation (CS), avoid AAF leakage, and avoid problematic retractor passage.	CS, VB	12	3	36
Perinate not reached in the uterus	The transfer bag may impede access for the operator to reach and grab the perinate.	CS, VB	12	2	24
Perinate cannot pass through retractor	The passage may be too small for the perinate if the retractor is overinflated or misaligned. If the retractor is underinflated, material collection may prevent the perinate from passing through (or cause the retractor to be pushed out).	VB	12	2	24
The device contains too less grip to hold the device properly	Due to the use of fluid, a slippery transferbag could become a hazard.	CS, VB	12	2	24
Retractor does not fit	The uterine retractor (CS) internal ring must be kept behind the uterus wall for the device to perform effectively. This prevents fluid leaking into the maternal abdomen, device movement, perinate trapping between the uterus and the retractor, and uterine wall incision tamponade. For VB, poor design or folding location in the birth canal might cause fluid leakage, device movement, trauma to maternal tissue, or possible entrapment of the perinate between the retractor and birth canal.	CS, VB	12	2	24
Transferbag instability during cannulation	During the cannulation the transferbag/UC should be held in a stable position to prevent cannula dislocation due to traction.	CS, VB	12	2	24
Entrapment of material (sleeve/glove) in transfer device (clips/connector)	Tearing of the material with resulting leakage or inability to clamp transferbag to retractors could lead to placing unwanted pressure on the device and the mother's abdomen.	CS	12	2	24
Temperature drops	To avoid hypothermia of the perinate, and potential fetal to neonatal transition, it is essential to keep the temperature constant.	CS, VB	12	2	24

All fall in the category unacceptable risk control/redesign required.

The potential adverse outcomes associated with preterm lung aeration, such as the risk of transition from fetal to neonatal circulation, underscore the importance of avoiding lung aeration during the transfer procedure. Other hazards identified included those related to ergonomics; the weight of the filled transferbag, decreased dexterity or grip when using the integrated gloves, inadequate prototype design, assembly or placement impairing the passageway for the perinate, temperature maintenance.

#### 3.4.3 Risk assessment

Based on the filtering of the risk values greater than 12, a top 10 of potential hazards was extracted (see Table 2). Please see Supplementary Tables S3A and S3B for Fault Tree Analyses of the causal relationships of device and human failures for CS and VB respectively. For the FMEA risk assessments, we refer to Supplementary Tables S4A and S4B.

### 3.5 Mitigation and evaluation through simulation

All critical hazards (Table 2) were addressed in the last phase of the study, during (liquid-based) simulations and expert interviews (P36-P50). Table 3 lists the mitigations used to reduce or eliminate the risks with highest criticality scores. Adjusting procedure instructions and protocol guidelines decreased several hazards in addition to design changes.

**Table 3:** Overview of ten potential design hazards and their risk mitigations.

Hazard	Mitigation (numbered in case of multiple iterations)		
Air contact	1	2	3
	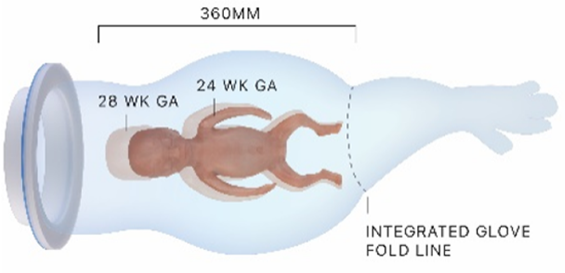	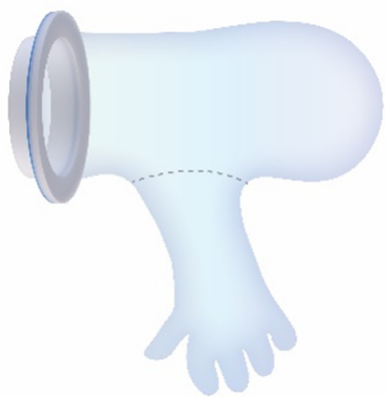	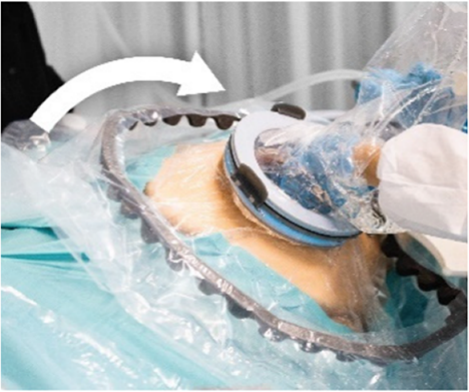
	(1) Transferbag of sufficient lenght for 24–28 week GA perinate. The integrated gloves are in laid-out geometry. (2) Transferbag with pouch to ensure bag is not emptied due to movement of glove (CS). (3) Change of maternal positioning for horizontal transfer. Other mitigations included rubber labyrinth seals of the transfer device parts (CS). Liquid simulation proved that action does not lead to a hazardous situation. However, time should be reserved during the procedure to release trapped air		
Transferbag too heavy	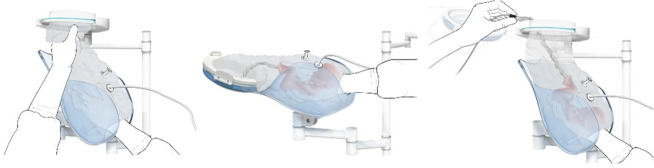		
	Transfer station and ring holder can be used during filling with delivery and cannulation to relieve weight		
Perinate is not reached/delayed delivery	Manikin was reached in every simulation		
Perinate cannot pass through retractor	1		2
	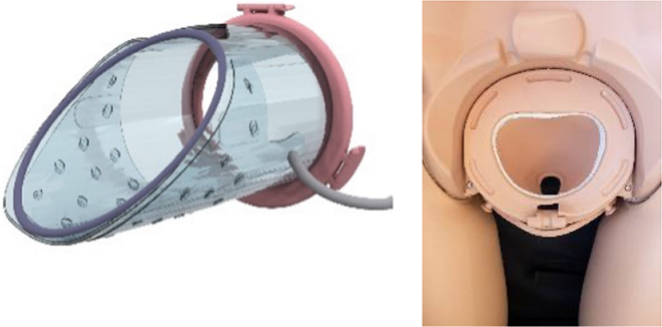 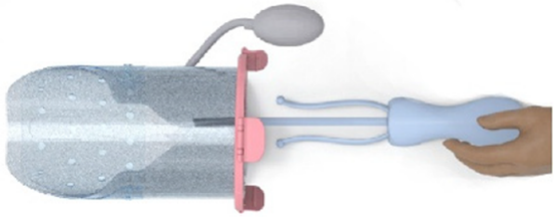		
	(1) Additional interior retraction ring allows for fixation around cervix (VB) and (2) a retractor applicator could be used to properly insert retractor (VB)		
Insufficient hand dexterity	1	2	3
	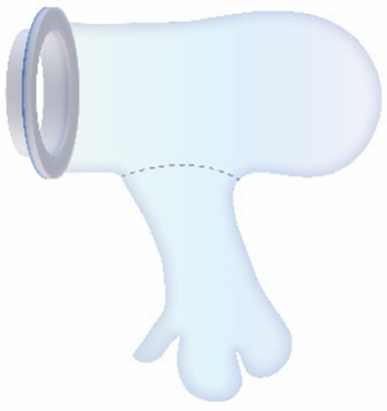 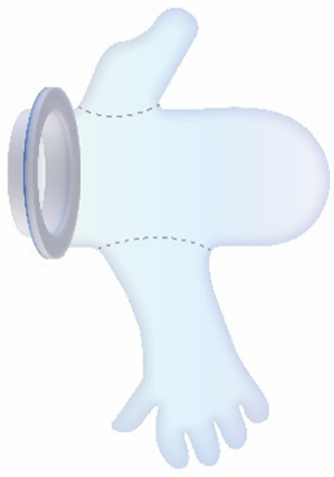 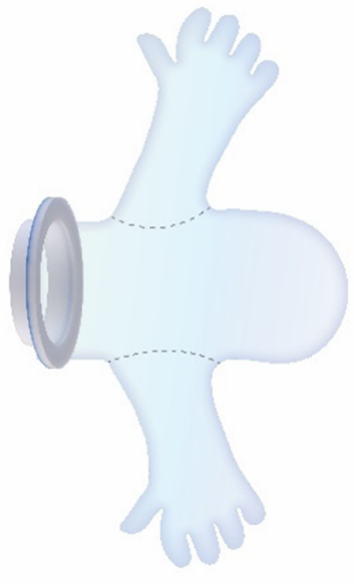		
	(1) Prototype with a 3-pocket integrated glove and (2) an additional 1-pocket integrated glove. (3) Final prototype of the transferbag contains two 5-finger integrated gloves (CS and VB)		
Insufficient grip	1	2	
	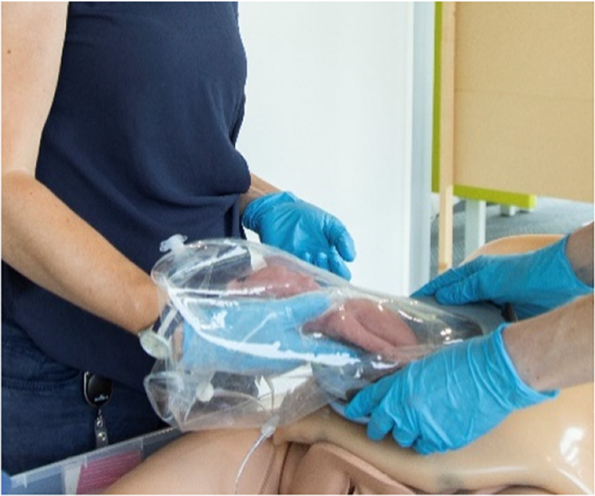	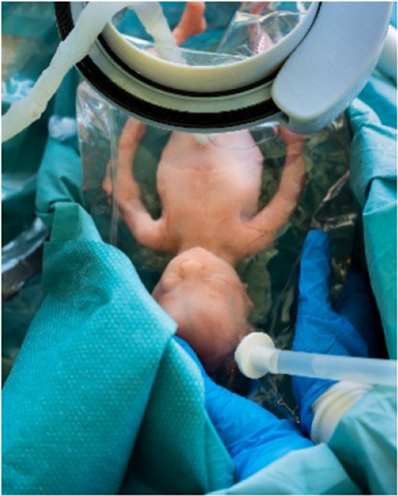	
	(1) Transferbag held by two clinicians. (2) Hazard could be mitigated using transferstation on which transferbag was placed		
Retractor does not fit	1	2	
	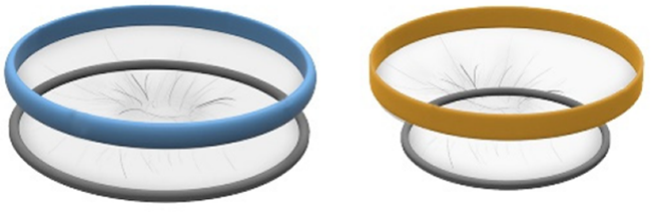	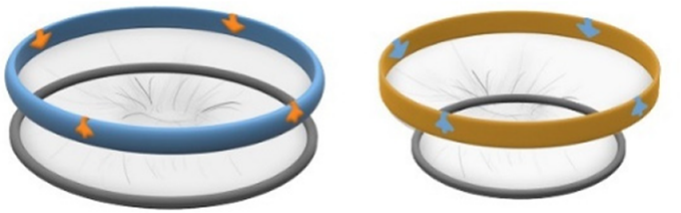	
	(1) 1st iteration retractors. (2) Additional design feature of the CS retractors with signs and color affordances to indicate direction of rolling for retraction. Left retractor shows the skin incision retractor, the right retractor is for the uterine incision		
Transferbag instability during cannulation	Hazard could be mitigated using transferstation (see design mitigation “insufficient grip”)		
Entrapment of material in transfer device	1	2	
	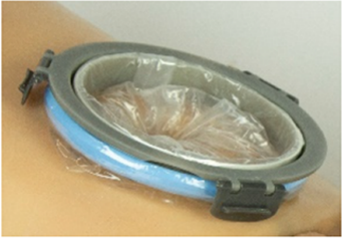 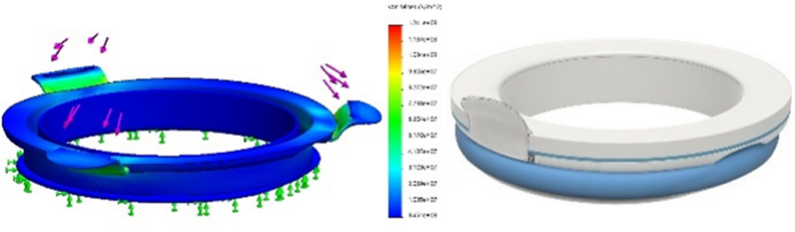		
	(1) Hinge-type clips. (2) Redesign of clips to allow quicker and easier attachment and release. Less hands needed due to snap-fit mechanism [finite element structural analysis (center)] (CS)		
Temperature drops	1	2	
	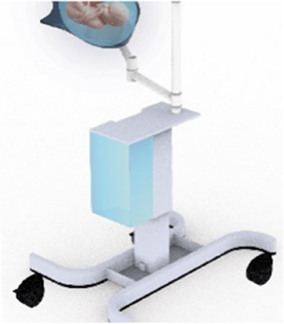	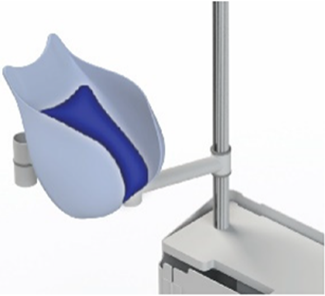	
	(1) 1st iteration of transfer station with a heated AAF reservoir. (2) Placement of warming pad in transferstation (Kent Scientific, USA)		

Continued submersion of the manikin's upper airways appeared difficult. To improve this, participants kept the transferbag horizontal throughout a CS simulation (P43–P47). Liquid-based simulations included placing the maternal manikin in 15° left lateral tilt, yet this proved insufficient. Fluid transferred from the bag into the native uterus when the hand entered the glove to reach for the manikin. Participants suggested positioning the glove in the center of the bag and making a fluid pouch at the transfer bag's end (P43–P47) (see Table 3). This design change made it easier to maintain the fluid volume but difficult to reach the manikin. Procedural adjustments included supplying extra fluid to the open uterine incision site; this amnioinfusion-like supply tube has again to be removed before attaching the transferbag. This can be facilitated when the surgical assistant controls the fluid supply and ensures that the fetal head remains submerged. Simulations showed that filling half of the transferbag before connection to the mother, allowed the perinatologist to comfortably put on the integrated glove. After connecting the transferbag to the device base, trapped air was released, additional fluid supplied, and the manikin was delivered under continuous submersion. To minimize dehydration and temperature decline, participants recommended keeping the perinate's temperature at the native uterus' temperature and the UC moist and warmed (P39, P41, P42).

Allowing placement of the manikin's whole body in the warmed liquid helped maintain its warmth (see Table 3). Design mitigation proposals for the transfer station were a warming pad onto which the transferbag can be placed and a continuously warmed liquid reservoir. Another aspect mentioned was the increased risk for infection in case the fragile skin gets into contact with the exterior, which was also addressed by the mitigation of full-body immersion (P39, P42).

**Figure 6:**
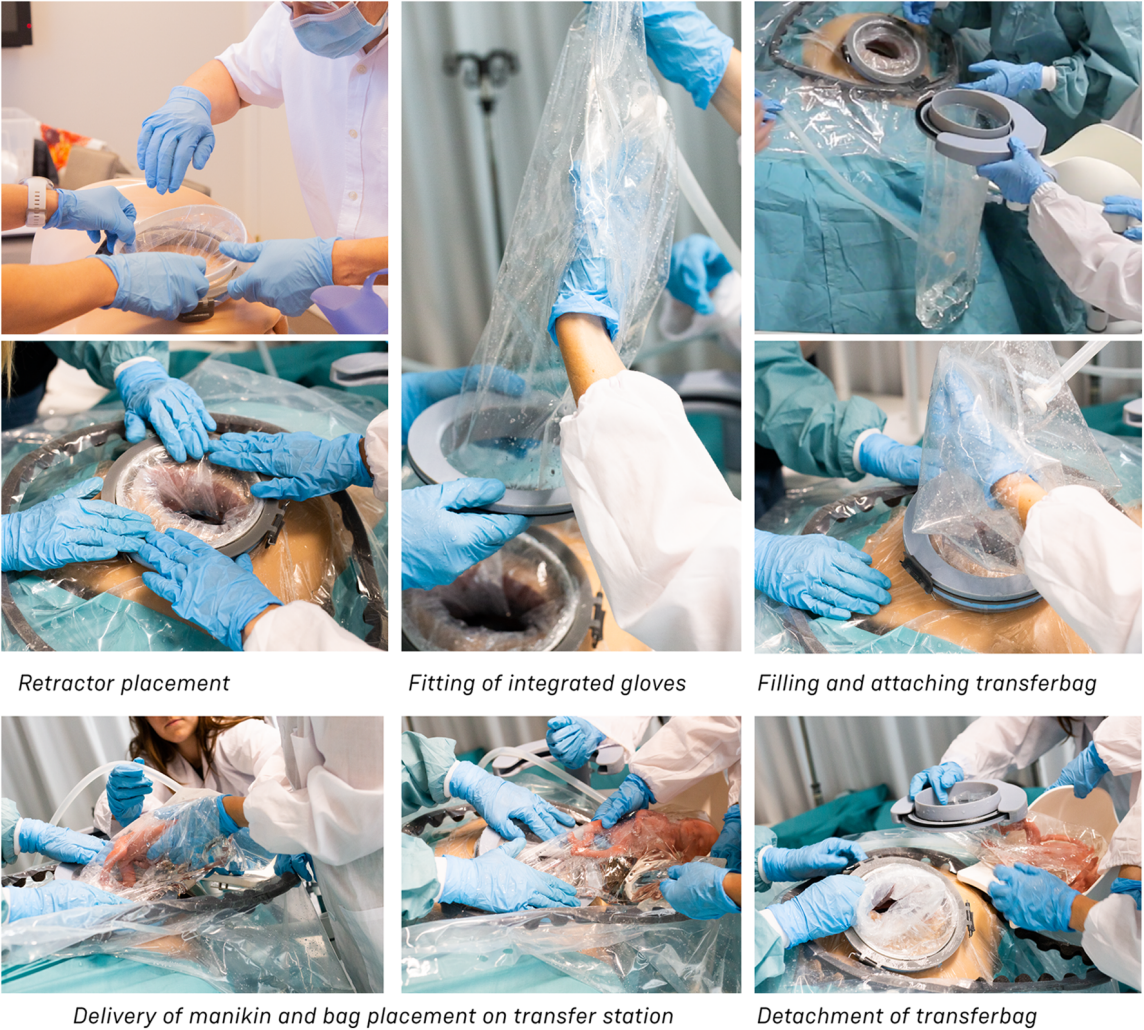
Multiple testing sessions using simulation. Shown: simulation session using the final transfer device prototypes.

At a certain point in the procedure, the transfer bag carried multiple liters of fluid and a manikin, which posed a risk of operator fatigue and an increased likelihood of dropping it. To alleviate this, a platform onto which the bag could be placed was proposed, i.e., a transferstation, and proved essential during subsequent simulations for bag handling, to stably place the manikin after delivery for examination (see Figures 5, 6). In the simulations, the transferbag was filled while supported vertically whereas during the transfer, the station was tilted horizontal, and back to vertical mode during cannulation. A separate holder for the transferbag ring prevents spilling out of AAF. The limited length of the UC will make it necessary to keep the perinate close to the mother during cannulation, especially in case of a bypass cannulation. Using flexible and moveable arms the transfer station could be placed close to the mother (see Figures 5, 6).

The hazardous situation, wherein the perinate is unreachable while having the transferbag attached on the retractor connector, may arise due to various errors such as the perinate's inability to pass through the retractor, inadequate hand dexterity or grip. In a VB transfer, inflation of the retractor could form a hazard when overinflated, or when the retractor is wrongly positioned in the birth canal and the perinate cannot pass through. Making this retractor using TPU film instead of silicone and embedding specific laminations provided expandability within required ranges. An additional retractor ring at the interior side of the sleeve that fits around the cervix was suggested to prevent the retractor from moving (P50). This additional retractor ring could be bent upon entry, and thus mimics the insertion of a NuvaRing, a GelPOINT V-Path or the cervical retractor by Whalen et al. (35). In one simulation session, it was noted that the retractor, while moving slightly along with the manikin, remains positioned in the birth canal without outward movement due to the fixation ring. Aside from its benefits in positioning, the additional retractor ring demonstrated another positive effect. During another simulation session it was observed that although the retractor could slightly move along with the manikin, the retractor remained fixed in the birth canal due to the fixation ring, preventing outward displacement. Another suggestion for improved positioning that was fabricated, and tested was to embed a two-finger integrated glove in the retractor film to facilitate insertion or employ an applicator with arms that glide in and out (see Table 3). Lastly, markers or other labeling on the sleeve or ring were suggested to visually guide positioning.

During a regular delivery, either CS or VB, the minimum dexterity needed for perinatologists is to deliver with one hand and hold the fetal armpits with the ring and index fingers. With the aim of making a one-size fits all integrated glove, participants tried out gloves with 2 pockets (mitten), 3 pockets, and 4 pockets (middle and ring finger joined). The mitten and three-finger glove appeared too small to deliver the manikin (P29–P35), while 4-pockets functioned better (P43–P49). Simulation participants preferred a 5-finger glove (P43-P49), making it essential to develop transferbags with varied glove sizes to accommodate all medical staff. Yet, this creates an additional problem when two gynecologists want to switch roles after the start of the delivery.

To facilitate a secure grasp on the manikin and to speed up the delivery, an extra integrated glove designed for the right hand was introduced on the opposite side of the transfer bag, thereby also rendering the bag suitable for use by both left and right-handed gynecologists (P48, P49). The indirect contact with the manikin and the slippery environment caused by using fluids made it difficult to get a proper grasp on the manikin during liquid-based simulations. Participants proposed texturing the integrated glove on the material side that faces the clinician, which could be embedded in future prototypes (P45–P47). To attach the transferbag to the connector, clips must be locked, and this required some time and force during the simulations. Also, retractor film can become entrapped. Snap-fit, turn-and-lock and other sealing mechanisms were proposed that may reduce forces, allow easier attachment, and speed up the process (see Table 3).

During several simulation rounds participants addressed sterility management and proposed that the transfer station could be easily supplied with sterile wrapping. When the transferbag is left open, either before or after deliver, a hatch placed over the transferbag (with an opening for the UC) could improve to keep the AAF sterile (P43–P47).

Procedural improvement and human-related error mitigation regarding task division were also evaluated. In the first set of simulations the main surgeon, i.e., perinatologist 1, made incisions while positioned at the right side of the patient. Perinatologist 2, positioned at the left, inserted the retractors and delivered the manikin into the transferbag. Participants felt uncomfortable since the main surgeon usually stands on the patient's left side during a CS. Participants suggested, tested, and favored the left side execute incisions and deliveries, while the right-side perinatologist inserts wound retractors (P43, P44). It was evident that two scrub nurses, one on either side of the table, were required to handle the transfer device's parts, fill the bag with AAF, and adjust the transfer station (P45–P47).

## 4 Discussion

This study presents an exploration of a transfer procedure for two delivery modes, by employing simulation-based participatory methods for procedure and prototype development. APAW animal studies have successfully shown the potential of extra-uterine life support for extremely premature infants. This research started to explore the development of an adapted obstetric procedure for human patients using manikins, approached from a design perspective. These obstetric procedures have not yet been carried out in animal studies, and if they would be tested, there will undoubtedly be deviations owing to anatomical differences. As a result, although the use of VB in conjunction with APAW treatment is still speculative, this research does provide insight on the obstetric elements involved. We hypothesized that the use of medical simulation elicits realistic user insights to guide and improve the development for obstetric tools, whilst also envisioning a training ground to prepare its future users.

The main objective of this study was to form an understanding, propose and test concepts of a method to deliver the perinate whilst being shielded from exterior influences such as air exposure, temperature shifts and sound, light, and tactile stimulation. Simulations confirmed that the fetal manikin could be submerged during the entire procedure. Through the development of proof-of-concept prototypes and subsequent investigation of potential hazards, procedural and design refinements improved latent conditions. Certain factors, such as light and sound exposure or time-related measurements need further testing, for which relevant sensors embedded in the manikin could be used. This approach facilitates the testing of more concepts for feasibility and usability, and can complement *in vivo* studies.

Although the prototypes developed in this study were fabricated to perform user tests and simulations, they are not medical devices. The outcomes of this study have only been evaluated through medical simulation. During the design process, the applicable medical device regulations (Regulation EU 2017/745) were followed to be able to approach medical grade prototypes. The use of simulation technology allowed us to address low occurrence/probability, but high-risk events in a safe setting. Nevertheless, translation of the outcomes of this study to a clinical setting would need animal and/or in-human studies to verify its feasibility, safety, and efficacy.

High fidelity medical simulation can mimic certain, but not all clinical aspects that might be involved. Within the boundaries of the present simulation, pharmacological and physiological effects on the perinate and maternal anatomical differences were not investigated. The hazard identification in this study was based on expert opinion and simulation and accounted for risks based on our present understanding. More research would be needed, through animal studies or clinical studies, to confirm e.g., the thresholds of air inhalation (and other factors that could trigger neonatal transition) and maintenance of sterility. While infection control and sterility were included in the design criteria, their assessment was not the primary focus of the present simulations, which may have led to a lack of awareness on this issue during the hazard identification. Theoretical considerations for sterility control have been described before (58), but because of the etiology for preterm delivery often being caused by intra-amniotic inflammation or infection, and preterm premature rupture of membranes, this should be an explicit part of future investigations. Adequate fresh supply and drain of AAF is necessary when the infant is placed in the final fluid reservoir. Although ultimately this would need to be investigated *in vivo*, enabling sterility monitoring within a simulation setting, for example testing infection control using swaps, could add value to the simulation. In the case of a CS transfer, it is possible that the perinate's body is not fully and continuously submerged in AAF. For a brief interval between the uterus incision and attaching the transferbag, the head might be exposed to air, which could be prevented by amnioinfusion. Preterm delivery is an unpredictable and urgent event. Preterm premature rupture of the membranes may render a vaginal transfer procedure unfeasible regarding the prevention of air exposure. Several strategies have been suggested to overcome this (58), but future *in vivo* studies should elucidate the effect of a brief exposure to air in relation to neonatal transition.

The current study demonstrated a transfer approach from delivery, up to cannulation of the umbilical vessels while the manikin is already ex-utero. The transfer station in this study allowed support during these processes and can host the most essential components, the oxygenator, and a heated AAF reservoir. The procedure and prototypes developed in this study can form the basis for future research on subsequent steps in the procedure (cannulation and placement in the APAW system) and forthcoming changes in the technology. In addition, further research should explore the integration of the transferbag within the APAW system, or whether it can solely be used for delivery and transport. While UC cannulation received less attention in this study's simulations, connecting the umbilical vessels to the oxygenator is crucial and should occur during delivery, in proximity to the mother and within restricted time. The transfer station described in this study offers a platform from which this could be undertaken. Additional research should explore their potential consequences on the design of the prototypes outlined in this study. An already existing option that aids in the delayed clamping of the umbilical cord, by keeping the infant close to the mother is the Concord Neonatal cradle (LUMC, The Netherlands). This solution entails a platform that can be placed in proximity to the mother with the use of a moveable arm. If the UC is of short length, in-utero cannulation should also be considered. Existing EXIT-to-ECMO procedures in neonates are performed with an exteriorized umbilical cord while half of the lower body remains in-utero during cannulation (59), this overcomes the risk of uterus involution and shearing of the placenta. Likely, some adaptation must be made to improve compatibility with the in this article described procedure.

Further research could be directed to improving the mechanical aspects of the inflatable retractor design. Although the current simulations were performed on a single maternal manikin, its dimensioning may induce retractor misfitting for some patient groups, therefore different sizes should be provided. Secondly, due to the low friction coefficient of the birth canal, the VB retractor's external facing film may need texturing to assure adequate fixation in the birth canal. Thirdly, improvement can be made by modifying the CS skin incision retractor to allow for bidirectional rolling of the exterior ring. Fourthly, VB and CS retractor films' inner linings could be lubricated to prevent skin friction and damage to the perinate (60) and improve the move towards the transferbag. The white waxy substance that can sometimes be found on the skin of fetuses, vernix caseosa, also forms a protective biofilm, reducing friction during delivery (61). In terms of fabrication, the diameter, frequency, and pattern of the laminated regions all have an impact on the inflation behavior, which could be further optimized in case the simulation device prototypes will be translated beyond medical simulation use.

Next to the design improvements, procedural adjustments were proposed and tested, such as the maternal positioning during a CS. The standard maternal position during a CS is 15° left lateral tilt, to avoid vena cava and aortic compression, which could result in hypotension and reduced oxygen delivery to the fetus. However, studies have shown that a 15° tilt is almost never achieved, due to feasibility (62). Especially during the CS transfer procedure, it is essential to position the mother such that it allows for an almost horizontal passageway from the native uterus to the transferbag. Hence, in case the transferbag is held vertically and filled with AAF, the fluid will flow out of the transferbag directly into the uterus. A proposal to use a 30° left lateral tilt was by some participants regarded as unfavorable due to instability of the patient on the operating table, and the surgical difficulties it would cause. Other opinions included that if using general anesthesia, a 30° position would be possible, and if practiced intensively the surgical difficulties could be overcome. An additional effect is that anesthetics could also suppress breathing and thereby avoid lung aeration. Another suggestion involves a temporary manual side just before the delivery, that can be reversed when the perinate is placed in the transfer bag. The latter turned out to be the most promising during simulations.

Research showed that emergency surgical care involves more reported system errors than non-emergency care (63), making an emergency procedure like the transfer procedure more susceptible to errors. In the operating room, surgeons and their teams often face high-stress situations, especially when demands exceed available resources, leading to acute mental stress (64). Procedure complexity, surgical complications, time constraints, equipment failure, multitasking, and distractions may stress operating room personnel and impair performance (65, 66). To reduce the mental workload and ensure that the procedure can be properly performed by clinicians, we aimed to develop a set of tools that is simple to assemble, use and disassemble with a minimum number of components. We assume that the similarity between the prototypes of the two delivery types, including the visual cues for assembly, would lead to decreased simulation training time and a decrease of potential human errors in performing the transfer. In addition, training through the medical simulation developed in this study, may help to be familiarized with the procedure and thereby increase self-confidence (67). In the Supplementary Tables S2A and S2B, tasks 2.2 and 2.5–2.7 show the most subtasks which could be argued to result in a higher workload. Still, this assumption needs to be further evaluated by performing simulations assessed using mental workload tests such as the SURG-Task Load Index (64) an adaptation of the NASA-Task Load Index (68).

Further optimalisation and training of the procedure can be enabled by using manikins with advanced functionalities (69), such as equipped with an aeration sensor in the upper airways, connected to computational biological models that can further calculate cascades of physiological effects (70). Such hybrid physical-digital twins could offer real-time data-based simulations.

If APAW technology proves successful in promoting infant survival, a potential consequence could be the shifting of the limit of viability. Because this limit is also linked to legislation regarding abortion in various regions, there should be continuous research on the legal consequences of APAW and its potential impact on women's rights (71). Research on medical ethics, legislations, guidelines for patient selection for clinical trials and informed consent is therefore indispensable, and the discussion and establishment of guidelines should run in parallel to the technical efforts (9, 29). Although we acknowledge the importance of researching the moral and ethical implications of this technology, this is not investigated within this study. In this present study, only clinicians were involved in the participatory simulations and expert interviews. Future studies however, could make use of the simulation method presented here, to facilitate discussions with patient advocates and a wider range of stakeholders to bring patient and care-taker well-being, ethical and human resource aspects into the risk assessment and guarantee a value-sensitive evaluation of the procedure (9, 29).

## Data Availability

The original contributions presented in the study are included in the article/Supplementary Material, further inquiries can be directed to the corresponding author.

## Footnotes

^1^https://www.iso.org/standard/72704.html.
