# Supplementary figures and images for: From intra- to extra-uterine: early phase design of a transfer to extra-uterine life support through medical simulation
Source: Front Med Technol. 2024 Aug 20;6:1371447. doi: 10.3389/fmedt.2024.1371447 (PMC11368740; doi:10.3389/fmedt.2024.1371447)

## Fault Tree Analysis - VAG Transfer Procedure

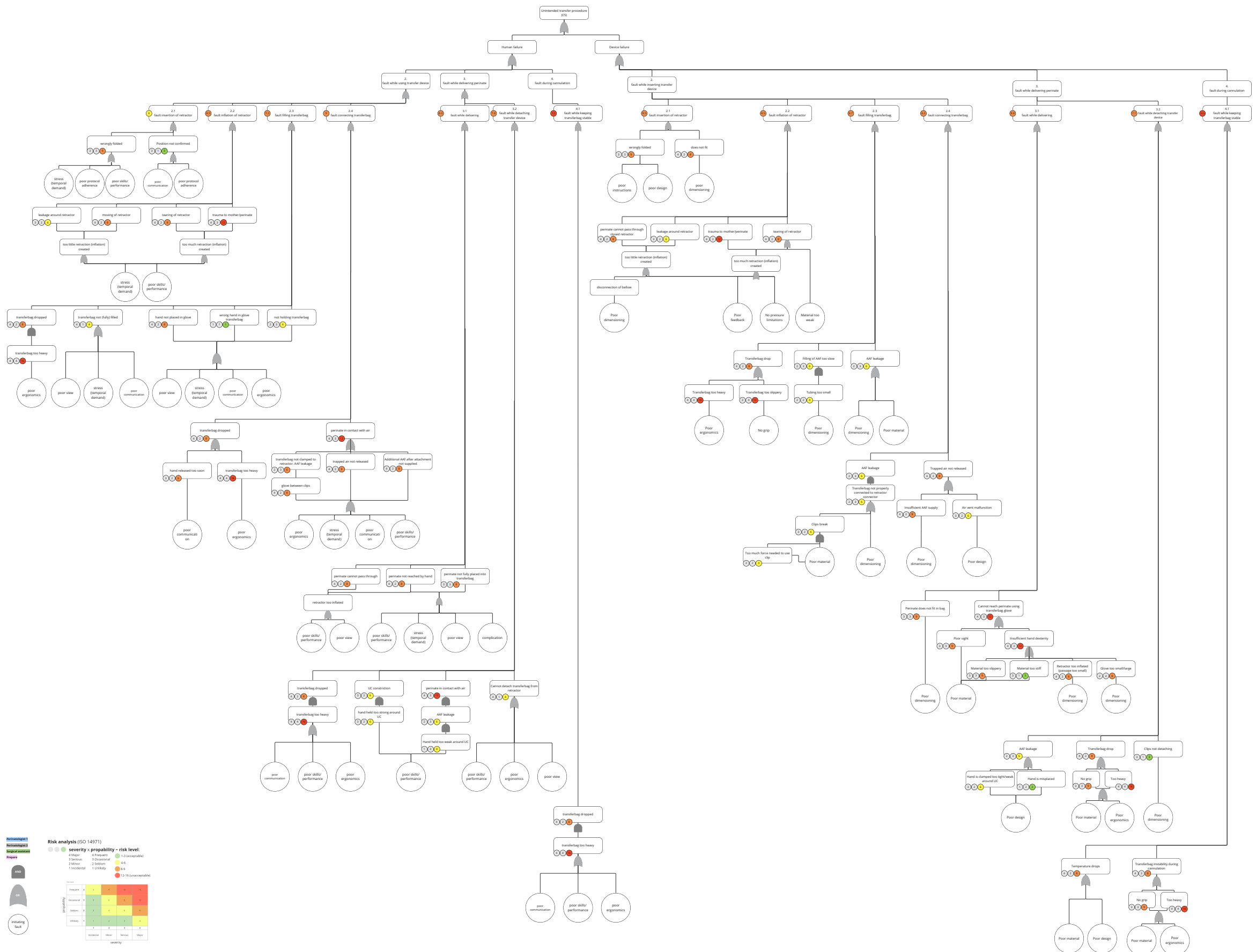

Supplement: Supplementary file 1 [file Datasheet1.pdf]

## Fault Tree Analysis - CS Transfer Procedure

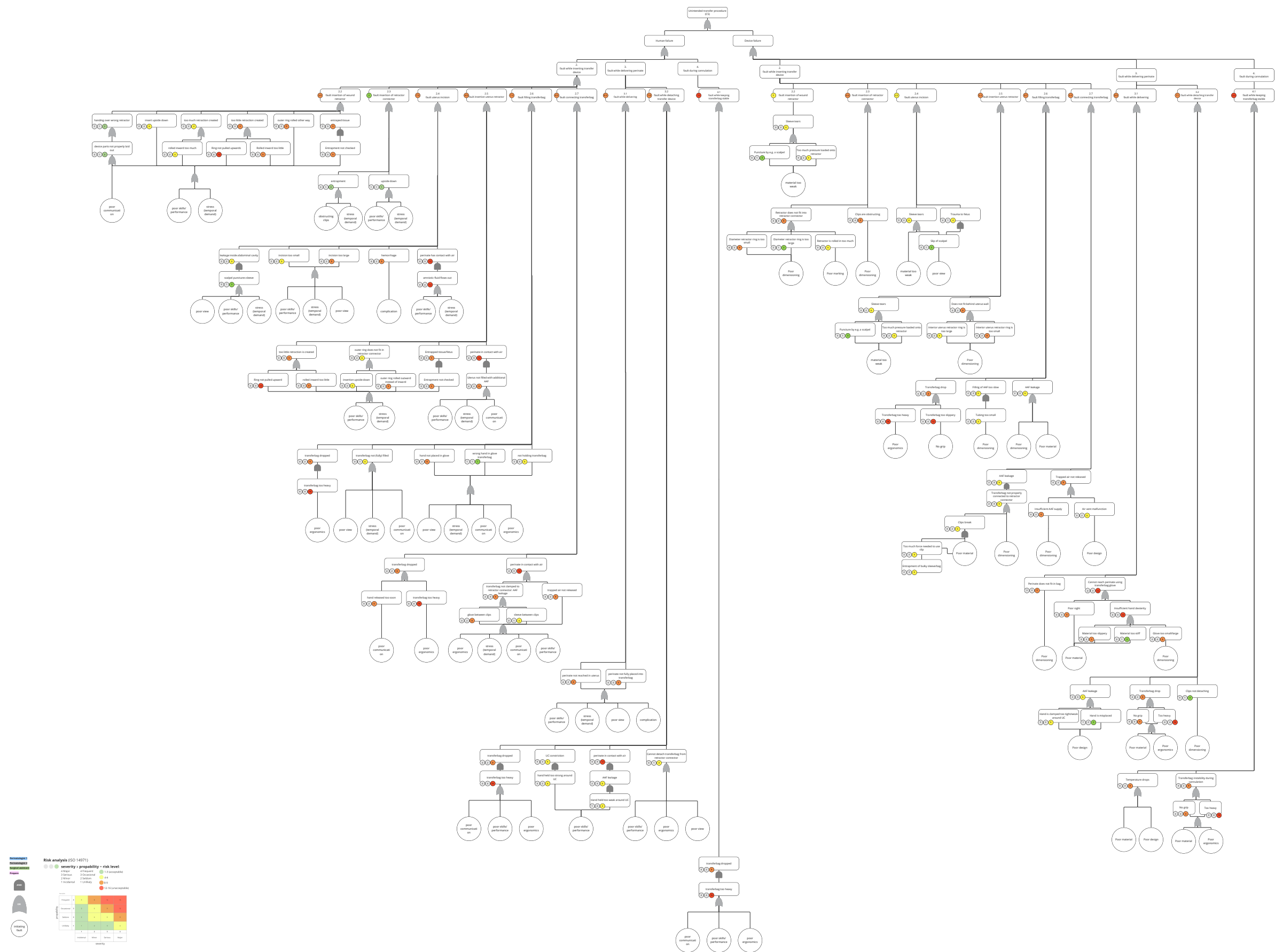

Supplement: Supplementary file 2 [file Datasheet2.pdf]
