# Supplementary material for: From intra- to extra-uterine: early phase design of a transfer to extra-uterine life support through medical simulation
Source: Front Med Technol. 2024 Aug 20;6:1371447. doi: 10.3389/fmedt.2024.1371447 (PMC11368740; doi:10.3389/fmedt.2024.1371447)
Supplement: Supplementary file 3 [file Datasheet3.pdf]

(red)	Unacceptable risk control/redesign required
(orange)	Risk control and investigation required
(yellow)	Little risk
(green)	Risk is acceptable and no risk control is required

ID #	Condition of failure	Main tasks	Potential failure mode (hazzard)	Potential causes	Hazardous situation	Potential harmful effects	SEV	PROP	RPN	SEV	PROP	RPN	Risk control measures
7	Human	2.3	Transferbag too heavy	Task time too long, transferbag contains too much liquid, not enough hands/support available	Operator drops transferbag	(Lethal) trauma, psychological damage to parents, ergonomic problems operators, procedure cancelled	4	4	16	12	4	48	Minimize duration how long the operator needs to hold the filled transferbag before delivery. Provide support during transfer procedure.
13	Human	2.4	Transferbag too heavy	Task time too long, transferbag contains too much liquid, not enough hands/support available	Operator drops transferbag	(Lethal) trauma, psychological damage to parents, ergonomic problems operators,cancellation of procedure	4	4	16	12	4	48	Minimize duration how long the operator needs to hold the filled transferbag before delivery. Provide support during transfer procedure.
28	Human	3.2	Transferbag too heavy	Task time too long, transferbag contains too much liquid, not enough hands/support available	Operator drops transferbag	(Lethal) trauma, psychological damage to parents, ergonomic problems operators	4	4	16	12	4	48	Make sure how long the operator need to hold the filled transferbag before delivery. Provide support during transfer procedure.
32	Human	4.1	Transferbag too heavy	Task time too long, transferbag contains too much liquid, not enough hands/support available	Operator drops transferbag	(Lethal) trauma, psychological damage to parents, ergonomic problems operators	4	4	16	12	4	48	Minimize duration how long the operator needs to hold the filled transferbag before delivery. Provide support during transfer procedure.
40	Product	2.3	Transferbag too heavy	Task time too long, transferbag contains too much liquid, not enough hands/support available	Operator drops transferbag	(Lethal) trauma, psychological damage to parents, ergonomic problems operators	4	4	16	12	4	48	Minimize duration how long the operator needs to hold the filled transferbag before delivery. Provide support during transfer procedure.
63	Product	3.2	Transferbag too heavy	Task time too long, transferbag contains too much liquid, not enough hands/support available	Operator drops transferbag	(Lethal) trauma, psychological damage to parents, ergonomic problems operators	4	4	16	12	4	48	Minimize duration how long the operator needs to hold the filled transferbag before delivery. Provide support during transfer procedure.
69	Product	4.1	Transferbag too heavy	Task time too long, transferbag contains too much liquid, not enough hands/support available	Operator drops transferbag	(Lethal) trauma, psychological damage to parents, ergonomic problems operators	4	4	16	12	4	48	Minimize duration how long the operator needs to hold the filled transferbag before delivery. Provide support during transfer procedure.
6	Human	2.2	trauma to mother/perinate	Too much retraction (inflation) created, stress, poor skills/performance	Too much pressure on tissues, no quick access to trauma (retractor needs to be removed)	Cancellation of procedure	4	3	12	12	3	36	Improve material strength, improve design of retractor and use correct pressure needed. Level of pressure used should be safe
17	Human	2.4	Perinate in contact with air	Transferbag not properly attached, trapped air in bag, leakage	Breathing reflex activated	Air exposure, cancellation of procedure	4	3	12	12	3	36	Indicate to participants that it crucial to keep the perinate submerged in fluid.
19	Human	2.4	Trapped air not released	Poor communication, wrong guidance, poor procedure performance /skills, stress, vent malfunction, poor view	Perinate can get exposed to air	Air exposure, cancellation of procedure	4	3	12	12	3	36	Provide guidance of when and how to vent the air out of the transferbag. An indication to make clear how and where to vent out the air. Improve transparency material.
24	Human	3.2	Perinate in contact with air	Transferbag not properly attached, trapped air in bag, leakage	Breathing reflex activated	Air exposure, procedure cancelled: start of rescue procedure	4	3	12	12	3	36	Indicate to participants that it crucial to keep the perinate submerged in fluid.
38	Product	2.2	trauma to mother/perinate	Too much retraction (inflation) created, poor feedback mechanism, no pressure limitations	Too much pressure on tissues, no quick access to trauma (retractor needs to be removed)	Cancellation of procedure	4	3	12	12	3	36	Improve material strength, improve design of retractor and use correct pressure needed. Level of pressure used should be safe
57	Product	3.1	Insufficient hand dexterity	Poor material choices, poor dimensioning, too much pressure	Extended duration of the procedure, trauma to perinate if wrongly held, difficult to reach perinate, decreased dexterity	Trauma to perinate, extended duration, procedure cancelled	4	3	12	12	3	36	Ensure that the hand of the operator can smoothly enter the uterus (e.g. material, thickness, stiffness).
4	Human	2.2	moving of retractor	Too little retraction (inflation) created, forgotten to inflate, stress, poor skills/performance	AAF leakage, retractor can become wrongly positioned and cause trauma, retractor might fall out during contractions	Slippery/unstable environment, perinate in contact with air	4	2	8	12	2	24	Improve training and protocol, improve design of retractor and use correct pressure needed, might need to keep retractor in birth canal using hand or device (during contractions)
5	Human	2.2	tearing of retractor	Too much retraction (inflation) created, stress, poor skills/performance	Too much pressure on (maternal) tissue, leakage	Perinate in contact with air,	4	2	8	12	2	24	Improve material strength, improve design of retractor and use correct pressure needed

8	Human	2.3	Transferbag dropped	Not enough grip, too slippery environment, no support, too heavy, poor communication, stress, poor view	Trauma, leakage, stretching of UC	(Lethal) trauma, psychological damage to parents, ergonomic problems operators, procedure cancelled	4	2	8	12	2	24	The transferbag is easy to hold and there must be prevented that the transferbag can slip out of the operators hand (e.g. handles, grip).
10	Human	2.3	Hand not placed in glove	Wrong guidance, poor procedure performance /skills, stress	When hand is not placed in glove and transferbag is already fully filled, excess liquid would need to be suctioned or it will flow into direction of infant	Too much force applied into birth canal	4	2	8	12	2	24	Clear guidance (first hand then filling of transferbag). Liquid suctioning device should be available.
14	Human	2.4	Transferbag dropped	Not enough grip, too slippery environment, no support, too heavy, poor communication, stress, poor view	Trauma, leakage, stretching of UC	(Lethal) trauma, psychological damage to parents, ergonomic problems operators, cancellation of procedure	4	2	8	12	2	24	The transferbag should be easy to hold and it must be prevented that the transferbag can slip out of the operators hand (e.g. handles, grip). Additional support necessary
18	Human	2.4	Glove between clips	Poor view, poor skills/performance, stress	Glove get stuck, glove tears, leakage, procedure is longer, new transferbag needs to be used	Infection, procedure time longer, air exposure	4	2	8	12	2	24	Adjust the clips in a way that the connection of the transferbag to the device ring is easy to attach and do not bother the operator.
20	Human	2.4	Additional AAF after attachment not supplied	poor protocol adherence, poor communication, poor procedure performance /skills, stress, poor ergonomics	Perinate can get exposed to air	Air exposure, cancellation of procedure	4	2	8	12	2	24	Provide guidance of when and how to vent the air out of the transferbag. An indication to make clear how and where to vent out the air. And when to use AAF supply. Improve transparency material.
21	Human	3.1	Perinate not reached by hand	Passage through retractor too small (too inflated), not enough dexterity in bag, poor view, poor skills, outer retractor ring forms an obstacle	Procedure is longer, more force is required, retractor needs to be deflated	Tissue trauma, procedure time longer	4	2	8	12	2	24	Training needed for understanding dexterity in transferbag. Transferbag glove design improvement.
23	Human	3.1	Perinate cannot pass through	Passage through retractor too small (too inflated), outer retractor ring forms an obstacle, breech position infant	Difficult or impossible to guide the infant through the birth canal	Trauma to perinate/mother, asphyxia when prolonged, cancellation of procedure	4	2	8	12	2	24	Transferbag redesign, different sizes of retractor needed, quick deflation mechanism necessary
29	Human	3.2	Transferbag dropped	Not enough grip, too slippery environment, no support, too heavy, poor communication, stress, poor view	Trauma, leakage, stretching of UC	(Lethal) trauma, psychological damage to parents, ergonomic problems operators	4	2	8	12	2	24	The transferbag should be easy to hold and there must be prevented that the transferbag can slip out of the operators hand (e.g. handles, grip).
33	Human	4.1	Transferbag dropped	Not enough grip, too slippery environment, no support, too heavy, poor communication, stress, poor view	Trauma, leakage, stretching of UC	(Lethal) trauma, psychological damage to parents, ergonomic problems operators	4	2	8	12	2	24	The transferbag should be easy to hold and it must be prevented that the transferbag can slip out of the operators hand (e.g. handles, grip). Additional support necessary
35	Product	2.1	does not fit	Poor dimensioning	Procedure cannot be performed, if it will still be used: not inflate properly (with kinks/bends), leakage perinate will not be able to pass through.	Leakage, trauma to mother/perinate, perinate cannot pass through, extended procedure duration, cancellation of procedure.	4	2	8	12	2	24	More sizes need to be available. Pre-birth, dimensions of birth canal patient need to be measured.
36	Product	2.2	Perinate cannot pass through closed retractor	Passage through retractor too small (too inflated), outer retractor ring forms an obstacle, breech position infant	Difficult or impossible to guide the infant through the birth canal	Trauma to perinate/mother, asphyxia when prolonged, cancellation of procedure	4	2	8	12	2	24	Transferbag redesign, different sizes of retractor needed, quick deflation mechanism necessary
39	Product	2.2	tearing of retractor	Too much retraction (inflation) created, poor feedback mechanism, no pressure limitations	Too much pressure on (maternal) tissue, leakage	Perinate in contact with air,	4	2	8	12	2	24	Improve material strength, improve design of retractor and use correct pressure needed
41	Product	2.3	Transferbag dropped	Not enough grip, too slippery environment, no support, too heavy, poor communication, stress, poor view	Trauma, leakage, stretching of UC	(Lethal) trauma, psychological damage to parents, ergonomic problems operators	4	2	8	12	2	24	The transferbag should be easy to hold and it must be prevented that the transferbag can slip out of the operators hand (e.g. handles, grip). Additional support necessary
49	Product	2.4	Trapped air not released	Air vent is not working properly, vent is not releasing quick enough	Procedure takes longer, infant comes in contact with air	Procedure takes longer to allow air to be released (too long a procedure endangers the fetus because UC needs to be cannulated), air exposure	4	2	8	12	2	24	An indication to make clear how and where to vent out the air.
52	Product	2.4	Insufficient AAF supply	Tubing too small, too little pressure, leakage, AAF reservoir not sufficient	Extended preparation, extended procedure duration, becoming too heavy to hold the transferbag	Ergonomic issues, risk of dropping too heavy transferbag	4	2	8	12	2	24	Improve tubing dimensions. Connection tube to transferbag should be able to attain enough pressure. Improve AAF reservoir dimensions

54	Product	3.1	Cannot reach perinate using transferbag glove	Poor material choices (too stiff), poor dimensioning (glove should be elongated), not enough grip, not enough dexterity, poor view	Extended duration of the procedure, trauma to perinate if wrongly held	Trauma to perinate, extend duration, procedure cancelled	4	2	8	12	2	24	Ensure that the hand of the operator can smoothly enter the uterus (e.g. material, thickness, stiffness).
59	Product	3.1	Retractor too inflated (passage too small)	Passage through retractor too small (too inflated), forms an obstacle, breech position infant, deflation takes too slow or does not work	Difficult or impossible to guide the infant through the birth canal	Trauma to perinate/mother, asphyxia when prolonged, cancellation of procedure	4	2	8	12	2	24	Transferbag redesign, different sizes of retractor needed, quick deflation mechanism necessary
62	Product	3.2	Transferbag dropped	Not enough grip, too slippery environment, no support, too heavy	Trauma, leakage, stretching of UC	(Lethal) trauma, psychological damage to parents, ergonomic problems operators	4	2	8	12	2	24	The transferbag should be easy to hold and it must be prevented that the transferbag can slip out of the operators hand (e.g. handles, grip). Additional support necessary
68	Product	4.1	Temperature drops	Poor material choices, not enough AAF (e.g. because of leakage), procedure takes too long	Hypothermia	Rescue procedure	4	2	8	12	2	24	Enough AAF should be supplied to the transferbag, enough AAF should be available in reservoir, measures to maintain perinate at temperature should be sufficient.
71	Product	4.1	Transferbag instability during cannulation	Not enough grip, too slippery environment, no support, too heavy, poor view	Trauma, leakage, stretching of UC, cannula dislocation or rupture.	(Lethal) trauma, psychological damage to parents, ergonomic problems operators	4	2	8	12	2	24	The transferbag should be easy to hold and it must be prevented that the transferbag can slip out of the operators hand (e.g. handles, grip). Additional support necessary
9	Human	2.3	Transferbag not (fully) filled	Wrong guidance, poor procedure performance /skills, stress, poor view, poor communication	Perinate can be exposed to air, temperature drop	Air exposure, rescue procedure, hypothermia	4	1	4	12	1	12	Indicate to participants that it crucial to keep the perinate submerged in fluid. Material might need to be translucent for clear view.
30	Human	3.2	Cannot detach transferbag from retractor connector	Entrapment, poor procedure performance /skills, stress, broken clips, not enough hands available	Procedure takes longer, leakage, when prolonged: asphyxia of perinate	Procedure takes longer, danger of discontinuation of procedure	4	1	4	12	1	12	Adjust the clips in a way that the connection of the transferbag to the device ring is easy to attach and do not bother the operator.
42	Product	2.3	Transferbag too slippery	Slippery material, AAF leakage onto surgical space, no spots to hold the device	Operator might drop the transferbag or place too much pressure on perinate to grasp the bag	Trauma to the perinate, ergonomic/physical issues for the operator	3	4	12	3	4	12	The transferbag should be easy to hold and it must be prevented that the transferbag can slip out of the operators hand (e.g. handles, grip).
1	Human	2.1	wrongly folded	Unclear, poor protocol adherence, poor communication, poor procedure performance /skills, stress	Retractor would not fit in birth canal, retractor will not inflate properly (with kinks/bends), leakage perinate will not be able to pass through.	Trauma to mother/perinate, perinate cannot pass through, extended procedure duration, cancellation of procedure.	3	3	9	3	3	9	Clearly instruct how to insert the interior ring of retractor and prevent malpositioning. An indication of the upper and bottom side (e.g. color).Pre folded in package. Attached glove to insert properly.
22	Human	3.1	Perinate not fully placed in transferbag	Poor procedure performance /skills, stress, disturbed view, difficult to move the perinate inside the bag with one hand	Difficult or impossible to close the transferbag, leakage, temperature maintenance of perinate impaired	Hypothermia, trauma, leakage	3	3	9	3	3	9	Transferbag redesign, better grip within gloves, temperature maintenance assurance.
34	Product	2.1	wrongly folded	Poor instructions, poor design	Retractor would not fit in birth canal, retractor will not inflate properly (with kinks/bends), leakage perinate will not be able to pass through.	Trauma to mother/perinate, perinate cannot pass through, extended procedure duration, cancellation of procedure.	3	3	9	3	3	9	An indication of the upper and bottom side (e.g. color).Pre folded in package. Attached glove to insert properly. Or an applicator device to correctly position retractor.
53	Product	3.1	Perinate does not fit inside the bag	Poor material choices (too stiff), poor dimensioning	Extend duration of the procedure, too much force exerted to place infant in transferbag, breech presentation (perinate's head not in transferbag)	Trauma to perinate, air exposure if head is not brought into transferbag	3	3	9	3	3	9	Make sure that the perinate fits completely inside the transferbag but with enough space to clamp and close the bag. Different sizes of bags available.
56	Product	3.1	Material too slippery	Poor material choices, excessive fluid at the surgical site	Instability, transferbag could be dropped, perinate cannot be reached or slips out of hands gloves back into uterus	Trauma to perinate, extended duration, procedure cancelled	3	3	9	3	3	9	Make us of material that has enough grip on the surface.
60	Product	3.1	Poor sight	Accumulation of material, transferbag opacity, positioning of mother	Difficult to reach perinate, extended duration before reaching perinate (asphyxia risk), trauma to perinate due to unintended place of holding hand	Trauma to perinate, ergonomic/physical issues operator, extended duration of procedure, procedure cancelled	3	3	9	3	3	9	Ensure that material is transparent enough, reduce risk of material accumulation
61	Product	3.2	No grip	Slippery material, no places to hold the device, excessive fluid at site	Operator drops transferbag, extedned duration before reaching perinate, trauma to perinate (slips away and could hit parts of device)	Trauma to the perinate, ergonomic/physical issues for the operator	3	3	9	3	3	9	Make use of materials that have grip especially on the place where the operator holds the device.

70	Product	4.1	No grip	Slippery material, no places to hold the device, excessive fluid at surgical site	Operator drops transferbag, extedned duration before reaching perinate, trauma to perinate (slips away and could hit parts of device)	Trauma to the perinate, ergonomic/physical issues for the operator	3	3	9	3	3	9	Make use of materials that have grip especially on the place where the operator holds the device.
58	Product	3.1	Glove too small/large	Poor dimensioning, poor material choices (stiffness/flexibility)	material accumulation, risk of tearing the glove/transferbag, leakage, poor dexterity, difficult to reach perinate, extended duration of the procedure (transferbag might need to be changed	leakage, trauma to perinate, ergonomic/ physical issues for the operator	2	4	8	2	4	8	The glove should be comfortable for operators with small or large hands. Make multiple sizes of gloves available.
3	Human	2.2	leakage around retractor	Too little retraction (inflation) created, forgotten to inflate, wrongly positioned, stress, poor skills/performance	AAF leakage, air in transferbag, retractor might fall out during contractions	Perinate in contact with air, slippery environment	3	2	6	3	2	6	Improve training and protocol, improve design of retractor and use correct pressure needed
12	Human	2.3	Not holding transferbag	Wrong guidance, poor procedure performance /skills, stress	Too heavy for surgical assistant, procedure is longer	Ergonomic issues	2	3	6	2	3	6	It should be clearly indicated and trained how and when to hold and release the transferbag.
15	Human	2.4	Hand released too soon	Poor communication, wrong guidance, poor procedure performance /skills, stress	Dropping of transferbag	(Lethal) trauma, psychological damage to parents, ergonomic problems operators, leakage	3	2	6	3	2	6	Clear guidance and communication
16	Human	2.4	Transferbag not clamped to retractor:AAF leakage	Wrong guidance, poor procedure performance /skills, stress, poor communication, obstacle/entrapment, broken clips (too much force needed)	Leakage	Leakage, longer procedure duration, air exposure, rescue procedure	2	3	6	2	3	6	Clear guidance.
25	Human	3.2	Hand held too strong around UC	Wrong guidance, poor procedure performance /skills, stress, not clear how much pressure is used	Decreased UC blood flow	Physical damage to perinate	3	2	6	3	2	6	It should be clearly indicated how and when to clamp the transferbag. An clear and easy indication of how and where to close the transferbag around the umbilical cord (e.g. colors on the transferbag).
27	Human	3.2	AAF leakage	Wrong guidance, poor procedure performance /skills, stress, transferbag not quickly enough detached	Leakage, air entry into bag	Air exposure, rescue procedure	2	3	6	2	3	6	Ensure that the retractor ring fits tightto the transferbag ring to prevent movement or coming loose.
31	Human	3.2	UC constriction	Entrapment, poor procedure performance /skills, stress, not clear how much pressure is used	Trauma, when prolonged: asphyxia of perinate	Procedure takes longer, danger of discontinuation of procedure	3	2	6	3	2	6	Provide clear view and support for transferbag upper ring to be held upright.
37	Product	2.2	leakage around retractor	Too little retraction (inflation) created, disconnection of bellow, malfunctioning of bellow, material not airtight	AAF leakage, air in transferbag, retractor might fall out during contractions	Perinate in contact with air, slippery environment	3	2	6	3	2	6	Improve training and protocol, improve design of retractor and use correct pressure needed
44	Product	2.3	Filling of AAF too slow	Tubing too small, too little pressure, leakage	Extended preparation, extended procedure duration, becoming too heavy to hold the transferbag	Ergonomic issues, risk of dropping too heavy transferbag	2	3	6	2	3	6	Improve tubing dimensions. Connection tube to transferbag should be able to attain enough pressure.
45	Product	2.3	AAF leakage	Poor dimensioning, poor material (not stress resistant), transferbag not properly attached to connector	Leakage, air entry into bag	Air exposure, rescue procedure	2	3	6	2	3	6	Ensure proper dimensioning and material that can hold the weight of all the liquid
46	Product	2.4	Clips break	Poor material choices, poor dimensioning, too much pressure, entrapment	Leakage, air entry into bag, perinate can become trapped between transferbag and retractor, clinicians divert attention to holding transferbag tight instead of care for perinate	Longer duration of procedure, leakage, air exposure	3	2	6	3	2	6	Adjust the clips in a way that the connection of the transferbag to the retractor is easy to attach and do not bother the operator. Proper material choices and dimensioning
48	Product	2.4	Transferbag not properly connected to retractor connector	Wrong dimensioning, entrapment of sleeve/glove/tissue between two parts	Leakage, too much force exerted on tools and thereby mother's abdomen	Readjusting tools (procedure takes longer), air exposure, rescue procedure	2	3	6	2	3	6	Ensure that the device ring fits tight inside the outer ring of the wound retractor to prevent movement or coming loose.
50	Product	2.4	AAF leakage	Transferbag not properly attached, poor material of transferbag (cannot handle weight)	Leakage, air entry into bag	Air exposure, rescue procedure	2	3	6	2	3	6	Ensure that the device is sealed watertight (fabrication and material choices).
51	Product	2.4	Air vent malfunction	Too small, poor material choices, wrong placement of vent on transferbag (design choice)	Procedure takes longer: new transferbag might need to be prepared and attached	Procedure takes longer to allow air to be released (too long a procedure endangers the fetus because UC needs to be cannulated), air exposure	3	2	6	3	2	6	Make sure that the excess air in the transferbag can easily be removed but that air from outside cannot enter the transferbag.
65	Product	3.2	AAF leakage	Transferbag not properly (not quick enough) detached, poor material of transferbag (cannot handle weight)	Leakage, air entry into bag	Air exposure, rescue procedure	2	3	6	2	3	6	Ensure that the device is sealed watertight (fabrication and material choices).

66	Product	3.2	Hand clamped too tight/weak around UC	Poor view	Decreased UC blood flow, AAF can flow out and air can enter transferbag, leakage, perinate could fall out	Trauma to perinate	3	2	6	3	2	6	Moment of holding transferbag in this way should be minimized by offering support for transferbag.
26	Human	3.2	Hand held too weak around UC	Poor procedure performance /skills, stress, not clear how much pressure is used	AAF can flow out and air can enter transferbag, leakage, perinate could fall out	Physical damage to perinate, air exposure	1	4	4	1	4	4	It is clearly indicated how and when to clamp the transferbag. Perinate should be held with hand in glove of transferbag. An clear and easy indication of how and where to close the transferbag around the umbilical cord (e.g. colors on the transferbag).
43	Product	2.3	Tubing too small	Poor dimensioning	Extended preparation, extended procedure duration, becoming too heavy to hold the transferbag	Ergonomic issues, risk of dropping too heavy transferbag	2	2	4	2	2	4	Improve tubing dimensions.
47	Product	2.4	Too much force needed to use clip	Opertator applies too much pressure, (due to entrapment of material), stiff material of clip	Clips can break, too much force exerted on mother	Ergonomics, maternal trauma	2	2	4	2	2	4	Clip construction should be well dimensioned to resist forces exerted.
2	Human	2.1	Position not confirmed	Forget check, poor protocol adherence, poor procedure performance /skills stress, poor view	Retractor might be wrongly positioned, subsequent inflation can cause trauma	Tissue trauma mother/perinate, leakage, perinate cannot pass through, extended procedure duration	3	1	3	3	1	3	Improve protocol adherence, improve protocol. Position can be confirmed using markers for echo.
55	Product	3.1	Material too stiff	Poor material choices	Extended duration of the procedure, trauma to perinate if wrongly held, difficult to reach perinate, decreased dexterity	Trauma to perinate, extend duration, procedure cancelled	3	1	3	3	1	3	Make use of flexible, stiff, and impact-resistant materials.
64	Product	3.2	Clips not detaching	Poor material choices, entrapment	Extended duration of the procedure, force is applied and might cause trauma to mother or perinate, transferbag would need to be cut if clips are not detaching at all	Trauma to mother/perinate, ergonomic problems operators, procedure cancelled	3	1	3	3	1	3	Adjust the clips in a way that the connection of the transferbag to the device ring is easy to attach and do not bother the operator.
67	Product	3.2	Hand is misplaced (while detaching transferbag)	Poor view	Decreased UC blood flow, AAF can flow out and air can enter transferbag, leakage, perinate could fall out	Trauma to perinate	1	2	2	1	2	2	Markers on tranfserbag to show where hand should be placed while detaching transferbag from retractor connector.
11	Human	2.3	Wrong hand in transferbag	Wrong guidance, poor procedure performance /skills, stress	Difficult to deliver the perinate, longer procedure time	Longer procedure duration	1	1	1	1	1	1	An extra transferbag.
