# Supplementary material for: From intra- to extra-uterine: early phase design of a transfer to extra-uterine life support through medical simulation
Source: Front Med Technol. 2024 Aug 20;6:1371447. doi: 10.3389/fmedt.2024.1371447 (PMC11368740; doi:10.3389/fmedt.2024.1371447)
Supplement: Supplementary file 4 [file Datasheet4.pdf]

Red	Unacceptable risk control/redesign required
Orange	Risk control and investigation required
Yellow	Little risk
Green	Risk is acceptable and no risk control is required

ID #	Condition of failure	Main tasks	Potential failure mode (hazzard)	Potential causes	Hazardous situation	Potential harmful effects	SEV	PROP	RPN		SEV	PROP	RPN		Risk control measures
1	Human	2.2	Handing over wrong retractor	Unclear, wrong guidance, poor communication, poor procedure performance /skills, stress	Retractor is too small to fit retractor retractor	Longer procedure time (re-inserting correct retractor) and potential rupture of the uterus retractor when stretched	2	1	2		2	1	2		Set should be available in duplo. Clear color differences between retractors.
2	Human	2.2	Device parts are not laid out properly	Unclear, wrong guidance, poor communication, poor procedure performance /skills, stress	Usage of wrong instruments	Longer procedure time	3	1	3		3	1	3		Clear guidance/Instructions, feedback in communication
3	Human	2.2	Insert upside down	Unclear, wrong guidance, poor communication, poor procedure performance /skills, stress	Retractor would not fit retractor connector	Damage abdominal cavity, longer procedure time	3	2	6		3	2	6		Clearly instruct how to insert the interior ring and prevent entrapment. An indication of the difference between the inner and outer ring (e.g. color). An indication on the top of the device ring. Set should be available in duplo.
4	Human	2.2	Too much retraction created	Unclear, wrong guidance, poor communication, poor procedure performance /skills, stress	Retractor does not fit into retractor connector, too much pressure on maternal tissue, leakage	Damage, air exposure, start of rescue procedure	2	2	4		2	2	4		To prevent the operator from rolling the outer ring too little or too much instructions and/or information should be given about the correct amount of retraction onto the wound retractor
5	Human	2.2	Rolled inward too much	Unclear, wrong guidance, poor communication, poor procedure performance /skills, stress	Retractor does not fit into retractor connector, too much pressure on maternal tissue, leakage	Damage, air exposure, start of rescue procedure	2	2	4		2	2	4		An indication of the minimum and maximum required retraction on the retractors
6	Human	2.2	Too little retraction created	Unclear, wrong guidance, poor communication, poor procedure performance /skills, stress	Forgotten to pull the outer ring upwards, or rolled inward too little	Transferbag can move during procedure, leakage of AAF	3	3	9		3	3	9		An indication of the minimum and maximum required retraction on the retractors
7	Human	2.2	Ring not pulled upwards	Unclear, wrong guidance, poor communication, poor procedure performance /skills, stress	The sleeve accumulates, difficult to roll the sleeve inward, device does not fit tight and moves, leakage	Air exposure, rescue procedure	3	4	12		3	4	12		Indicate that the operator pulls the outer ring upwards before rolling inward
8	Human	2.2	Rolled inward too little	Unclear, wrong guidance, poor communication, poor procedure performance /skills, stress, material too stiff	Transferbag can move during the procedure, leakage	Air exposure, rescue procedure	3	3	9		3	3	9		Clearly instruct how to insert the inner ring and prevent entrapment
9	Human	2.2	Exterior ring rolled outward instead of inward	Wrong guidance poor procedure performance /skills stress	Device ring does not fit tight in the outer ring of the wound retractor, transferdevice not fit tight and moves, leakage	Air exposure, rescue procedure	3	3	9		3	3	9		Clear guides should inform operator that wound retractor must be rolled inward and the uterus retractor outward
10	Human	2.2	Entrapped tissue	Wrong guidance poor procedure performance /skills stress, poor view	(Bowel) tissue can be entrapped, could lead to unsufficient retraction, leakage	Tissue trauma, leakage	3	3	9		3	3	9		Clearly instruct how to insert the interior ring and prevent entrapment
11	Human	2.2	Entrapment not checked	Forget check, wrong guidance, poor procedure performance /skills stress, poor view	(Bowel) tissue can be entrapped, could lead to unsufficient retraction, leakage	Tissue trauma, leakage	3	3	9		3	3	9		Clearly instruct how to insert the interior ring and prevent entrapment
12	Human	2.3	Entrapment	Wrong guidance poor procedure performance /skills stress	Longer procedure time	Tissue trauma, damage to retractor connector clips	3	1	3		3	1	3		Clearly instruct how to insert the interior ring and prevent entrapment
13	Human	2.3	Upside down	Wrong guidance poor procedure performance /skills stress	Longer procedure time	Longer procedure time	1	1	1		1	1	1		Show how to insert the retractor connector
14	Human	2.4	Incision too small	Wrong guidance poor procedure performance /skills stress	Uterus retractor will move, or not fit, leakage	Air exposure, rescue procedure	2	2	4		2	2	4		The required incision size made clear And/or a guidance or tool to indicate the incision site
15	Human	2.4	Incision too large	Wrong guidance poor procedure performance /skills stress	Uterus retractor will move, or not fit, leakage	A moving retractor might damage tissue, unstable work area, air exposure, rescue procedure	4	2	8		12	2	24		The required incision size made clear and/or a guidance or tool to indicate the incision site
16	Human	2.4	Hemorrhage	Complication, wrong guidance, poor procedure performance /skills stress	Other procedure must be executed to reduce the bleeding	Fetal/maternal tissue trauma	4	2	8		12	2	24		Bleeding tamponade can be achieved by increased retraction by retractors.
17	Human	2.4	Amniotic fluid flows out	Incision too large, uterus contractions	Perinate gets into contact with air and breathing reflex is activated	Air exposure, rescue procedure	3	4	12		3	4	12		Indicate to participants that it crucial to keep the perinate submerged in fluid. Supply fluid to incision site after incision.
18	Human	2.4	Perinate gets into contact with air	Incision too large, uterus contractions, wrong guidance, poor procedure performance	Perinate gets into contact with air and breathing reflex is activated	Air exposure, procedure cancelled: start of rescue procedure	4	3	12		12	3	36		Indicate to participants that it crucial to keep the perinate submerged in fluid. Supply fluid to incision site after incision.
19	Human	2.4	Leakage inside abdominal cavity	Scalpel punctures sleeve	Sleeve is ruptured and amniotic fluid can flow out.	Tissue trauma, leakage, air exposure	2	2	4		2	2	4		Material of sleeve should be puncture resistant, clear view is necessary
20	Human	2.4	Scalpel punctures sleeve	Poor view, poor skills/performance, stress	Sleeve is ruptured and amniotic fluid can flow out.	Tissue trauma, leakage, air exposure	3	1	3		3	1	3		Material of sleeve should be puncture resistant, clear view is necessary
21	Human	2.5	Too little retraction created	Poor view, wrong guidance, poor procedure performance /skills, stress	Channel between uterus and transferbag is not closed, moves during procedure, leakage	Tissue trauma, air exposure, rescue procedure	3	3	9		3	3	9		To prevent the operator from rolling the outer ring too little or too much instructions and/or information is given about the correct amount of retraction onto the wound retractor
22	Human	2.5	Retractor ring not pulled upwards	Poor procedure performance /skills, stress, obstacle	Sleeve accumulates, difficult to roll sleeve inward, transferdevice not tighty fitting, can move, leakage	Tissure trauma, air exposure	4	3	12		12	3	36		Indicate that the operator pulls the outer ring upwards before rolling inward
23	Human	2.5	Retractor ring rolled inward too little	Poor procedure performance /skills, stress, material too stiff	Retractor can move during procedure	Leakage into abdomen, air exposure, rescue procedure	3	3	9		3	3	9		An indication of the minimum and maximum required retraction on the retractors
24	Human	2.5	Insertion upside down	Wrong guidance, poor procedure performance /skills, stress	Exterior retractor ring is too large for uterus, interior retractor ring is too small to fit retractor connector	Longer procedure time, tissue trauma (uterus), rupture of retractor due to streching.	3	2	6		3	2	6		An indication of the minimum and maximum required retraction on the retractors An extra uterus retractor
25	Human	2.5	Exterior ring rolled outward instead of inward	Wrong guidance, poor procedure performance /skills, stress	Ensemble of tools will not fit tight	Leakage	3	3	9		3	3	9		Clear guides inform the operator that the wound retractor must be rolled inward and the uterus retractor outward
26	Human	2.5	Exterior ring does not fit inside the retractor connector	Wrong guidance, poor procedure performance /skills, stress, retractor upside down	Too much pressure loaded on the abdomen	Maternal tissue trauma	3	2	6		3	2	6		Clear guidance
27	Human	2.5	Entrapped tissue/fetus	Check forgotten, wrong guidance, poor procedure performance /skills, stress, poor view, complications	(Bowel) tissue and/or fetus can be entrapped, trauma, hemorrhage, upon release unsufficient retraction, leakage	Fetal/maternal tissue trauma, insufficient fit of device ensemble	3	3	9		3	3	9		Clear guidance/reminder

28	Human	2.5	Perinate in contact with air	Incision too large, uterus contractions, wrong guidance, poor procedure performance /skills, stress, wrong insertion uterus retractor	Breathing reflex activated	Air exposure, procedure cancelled: start of rescue procedure	4	3	12		12	3	36		Indicate to participants that it crucial to keep the perinate submerged in fluid. Supply fluid to incision site after incision.
29	Human	2.5	Entrapment not checked	Check forgotten, wrong guidance, poor procedure performance /skills, stress, poor view	(Bowel) tissue and/or fetus can be entrapped, trauma, hemorrhage, upon release unsufficient retraction, leakage	Fetal/maternal tissue trauma, insufficient fit of device ensemble	3	3	9		3	3	9		Clear guidance/reminder. Blood accumulation could have led to decreased view, suction whilst supplying additional AAF.
30	Human	2.5	Uterus not filled with additional AAF	Wrong guidance, poor procedure performance /skills, stress	Perinate exposed to air	Air exposure, rescue procedure	4	2	8		12	2	24		Indicate to participants that it crucial to keep the perinate submerged in fluid. Supply fluid to incision site after incision.
31	Human	2.6	Transferbag too heavy	Task time too long, transferbag contains too much liquid, not enough hands/support available	Operator drops transferbag	(Lethal) trauma, psychological damage to parents, ergonomic problems operators, procedure cancelled	4	4	16		12	4	48		Minimize duration how long the operator needs to hold the filled transferbag before delivery. Provide support during transfer procedure.
32	Human	2.6	Transferbag dropped	Not enough grip, too slippery environment, no support, too heavy, poor communication, stress, poor view	Trauma, leakage, stretching of UC	(Lethal) trauma, psychological damage to parents, ergonomic problems operators, procedure cancelled	4	2	8		12	2	24		The transferbag is easy to hold and there must be prevented that the transferbag can slip out of the operators hand (e.g. handles, grip).
33	Human	2.6	Transferbag not (fully) filled	Wrong guidance, poor procedure performance /skills, stress, poor view, poor communication	Perinate can be exposed to air, temperature drop	Air exposure, rescue procedure, hypothermia	4	1	4		12	1	12		Indicate to participants that it crucial to keep the perinate submerged in fluid. Supply fluid to incision site after incision. Material might need to be translucent for clear view.
34	Human	2.6	Hand not placed in glove	Wrong guidance, poor procedure performance /skills, stress	When hand is not placed in glove and transferbag is already fully filled, excess liquid would need to be suctioned or it will flow into maternal abdomen.	Too much force applied into uterus	4	2	8		12	2	24		Clear guidance (first hand then filling of transferbag). Liquid suctioning device should be available.
35	Human	2.6	Wrong hand in transferbag	Wrong guidance, poor procedure performance /skills, stress	Difficult to deliver the perinate, longer procedure time	Longer procedure duration	1	1	1		1	1	1		An extra transferbag.
36	Human	2.6	Not holding transferbag	Wrong guidance, poor procedure performance /skills, stress	Too heavy for surgical assistant, procedure is longer	Ergonomic issues	2	3	6		2	3	6		It should be clearly indicated and trained how and when to hold and release the transferbag.
37	Human	2.7	Transferbag too heavy	Task time too long, transferbag contains too much liquid, not enough hands/support available	Operator drops transferbag	(Lethal) trauma, psychological damage to parents, ergonomic problems operators, procedure cancelled	4	4	16		12	4	48		Minimize duration how long the operator needs to hold the filled transferbag before delivery. Provide support during transfer procedure.
38	Human	2.7	Transferbag dropped	Not enough grip, too slippery environment, no support, too heavy, poor communication, stress, poor view	Trauma, leakage, stretching of UC	(Lethal) trauma, psychological damage to parents, ergonomic problems operators, procedure cancelled	4	2	8		12	2	24		The transferbag should be easy to hold and it must be prevented that the transferbag can slip out of the operators hand (e.g. handles, grip). Additional support necessary
39	Human	2.7	Hand released too soon	Poor communication, wrong guidance, poor procedure performance /skills, stress	Dropping of transferbag	(Lethal) trauma, psychological damage to parents, ergonomic problems operators, leakage	3	2	6		3	2	6		Clear guidance and communication
40	Human	2.7	Transferbag not clamped to retractor connector	Wrong guidance, poor procedure performance /skills, stress, poor communication, obstacle/entrapment, broken clips (too much force needed)	Leakage	Leakage, longer procedure duration, air exposure, rescue procedure	2	3	6		2	3	6		Clear guidance.
41	Human	2.7	Perinate in contact with air	Transferbag not properly attached, trapped air in bag, leakage	Breathing reflex activated	Air exposure, procedure cancelled: start of rescue procedure	4	3	12		12	3	36		Indicate to participants that it crucial to keep the perinate submerged in fluid. Supply fluid to incision site after incision.
42	Human	2.7	Glove between clips	Poor view, poor skills/performance, stress	Glove get stuck, glove tears, leakage, procedure is longer, new transferbag needs to be used	Infection, procedure time longer, air exposure	4	2	8		12	2	24		Adjust the clips in a way that the connection of the transferbag to the device ring is easy to attach and do not bother the operator.
43	Human	2.7	Trapped air not released	Poor communication, wrong guidance, poor procedure performance /skills, stress, vent malfunction, poor view	Perinate can get exposed to air	Air exposure, rescue procedure	4	3	12		12	3	36		Provide guidance of when and how to vent the air out of the transferbag. An indication to make clear how and where to vent out the air.
44	Human	2.7	Sleeve between clips	Poor communication, wrong guidance, poor procedure performance /skills, stress	Sleeve gets stuck, sleeve tears, leakage	Infection, procedure time longer, air exposure, rescue procedure	4	1	4		12	1	12		Adjust the clips in a way that the connection of the transferbag to the device ring is easy to attach and do not bother the operator.
45	Human	3.1	Perinate not reached in uterus	Incision too small, not enough dexterity in bag, poor view, poor skills, retractor rings form obstacles	Procedure is longer, more force is required	Tissue trauma	4	2	8		12	2	24		Training needed for understanding dexterity in transferbag. Transferbag glove design improvement.
46	Human	3.1	Perinate not fully placed in transferbag	Poor procedure performance /skills, stress, disturbed view, difficult to move the perinate inside the bag with one hand	Difficult or impossible to close the transferbag, leakage, temperature maintenance of perinate impaired	Hypothermia, trauma, leakage	3	3	9		3	3	9		Transferbag redesign, better grip within gloves, temperature maintenance assurance.
47	Human	3.2	Perinate in contact with air	Transferbag not properly attached, trapped air in bag, leakage	Breathing reflex activated	Air exposure, procedure cancelled: start of rescue procedure	4	3	12		12	3	36		Indicate to participants that it crucial to keep the perinate submerged in fluid. Supply fluid to incision site after incision.
48	Human	3.2	Hand held too strong around UC	Wrong guidance, poor procedure performance /skills, stress, not clear how much pressure is used	Decreased UC blood flow	Physical damage to perinate	3	2	6		3	2	6		It should be clearly indicated how and when to clamp the transferbag. An clear and easy indication of how and where to close the transferbag around the umbilical cord (e.g. colors on the transferbag).
49	Human	3.2	Hand held too weak around UC	Poor procedure performance /skills, stress, not clear how much pressure is used	AAF can flow out and air can enter transferbag, leakage, perinate could fall out	Physical damage to perinate, air exposure	1	4	4		1	4	4		It is clearly indicated how and when to clamp the transferbag. Perinate should be held with hand in glove of transferbag. An clear and easy indication of how and where to close the transferbag around the umbilical cord (e.g. colors on the transferbag).
50	Human	3.2	AAF leakage	Wrong guidance, poor procedure performance /skills, stress, transferbag not quickly enough detached	Leakage, air entry into bag	Air exposure, rescue procedure	2	3	6		2	3	6		Ensure that the device ring fits tight inside the outer ring of the wound retractor to prevent movement or coming loose.
51	Human	3.2	Transferbag too heavy	Task time too long, transferbag contains too much liquid, not enough hands/support available	Operator drops transferbag	(Lethal) trauma, psychological damage to parents, ergonomic problems operators	4	4	16		12	4	48		Make sure how long the operator need to hold the filled transferbag before delivery. Provide support during transfer procedure.
52	Human	3.2	Transferbag dropped	Not enough grip, too slippery environment, no support, too heavy, poor communication, stress, poor view	Trauma, leakage, stretching of UC	(Lethal) trauma, psychological damage to parents, ergonomic problems operators	4	2	8		12	2	24		The transferbag should be easy to hold and there must be prevented that the transferbag can slip out of the operators hand (e.g. handles, grip).
53	Human	3.2	Cannot detach transferbag from retractor connector	Entrapment, poor procedure performance /skills, stress, broken clips, not enough hands available	Procedure takes longer, leakage, when prolonged: asphyxia of perinate	Procedure takes longer, danger of discontinuation of procedure	4	1	4		12	1	12		Adjust the clips in a way that the connection of the transferbag to the device ring is easy to attach and do not bother the operator.

54	Human	3.2	UC constriction	Entrapment, poor procedure performance /skills, stress, not clear how much pressure is used	Trauma, when prolonged: asphyxia of perinate	Procedure takes longer, danger of discontinuation of procedure	3	2	6		3	2	6	Provide clear view and support for transferbag upper ring to be held upright.
55	Human	4.1	Transferbag too heavy	Task time too long, transferbag contains too much liquid, not enough hands/support available	Operator drops transferbag	(Lethal) trauma, psychological damage to parents, ergonomic problems operators	4	4	16		12	4	48	Minimize duration how long the operator needs to hold the filled transferbag before delivery. Provide support during transfer procedure.
56	Human	4.1	Transferbag dropped	Not enough grip, too slippery environment, no support, too heavy, poor communication, stress, poor view	Trauma, leakage, stretching of UC	(Lethal) trauma, psychological damage to parents, ergonomic problems operators	4	2	8		12	2	24	The transferbag should be easy to hold and it must be prevented that the transferbag can slip out of the operators hand (e.g. handles, grip). Additional support necessary
57	Product	2.2	Sleeve tears	Material is too weak, too much pressure is loaded on the retractor, a sharp object touches the retractor	AAF can flow trough the sleeve, leakage inside abdominal cavity	Infection and leakage	3	2	6		3	2	6	An indication of the minimum and maximum required retraction on the retractors. Improved material that is puncture and tear resistant.
58	Product	2.2	Too much pressure loaded on the retractor	Wrong adherence to protocol, weak material	Too much force can lead to tissue trauma or rupture of the retractor	Tissue trauma, leakage	3	2	6		3	2	6	An indication of the minimum and maximum required retraction on the retractors. Improved material that is puncture and tear resistant.
59	Product	2.2	Puncture by e.g. scalpel	Weak material, poor view, poor skills	Sleeve tears, leakage in abdominal cavity, too less AAF in transferbag, trauma	Leakage, infection, trauma	3	1	3		3	1	3	Clearly instruct that no sharp object may touch the sleeve of the retractor to prevent rupturing. Improved material that is puncture and tear resistant.
60	Product	2.3	Diameter retractor ring is too small	Poor design, poor dimensioning, entrapment/obstacle, too much retraction	Leakage between the sleeve and device ring	Increased duration of procedure, air exposure	4	2	8		12	2	24	Ensure that the device ring fits tight inside the outer ring of the wound retractor to prevent movement or coming loose.
61	Product	2.3	Retractor does not fit into retractor connector	Poor design, poor dimensioning, entrapment/obstacle	Discontinue of procedure, leakage	Discontinue of procedure, leakage	4	2	8		12	2	24	Ensure that the device ring fits tight inside the outer ring of the wound retractor to prevent movement or coming loose.
62	Product	2.3	Diameter retrator ring is too large	Poor design, poor dimensioning, too little retraction	Procedure time is longer, damage to sleeve, excessive material can become entrapped	Increased duration of procedure, air exposrue	2	1	2		2	1	2	Ensure that the device ring fits tight inside the outer ring of the wound retractor to prevent movement or coming loose.
63	Product	2.3	Retractor is rolled in too much	Poor design, poor dimensioning, too much retraction	Damage to tissue, tearing of sleeve, cannot fit retractor connector	Tissue damage	3	2	6		3	2	6	An indication of the minimum and maximum required retraction on the retractors.
64	Product	2.3	Clips are obstructing	Poor design	Difficult to insert the device ring into the outer ring	Procedure is longer	2	4	8		2	4	8	Show how to insert the device ring. And improve the lock mechanism to prevent burden of the clips.
65	Product	2.4	Slip of scalpel	Poor view, too much exposure and too little protection of the sleeve	Sleeve tears, leakage in abdominal cavity, potential trauma to fetus	Air exposure, leakage, longer duration of procedure (needs to be redone)	3	1	3		3	1	3	Improved material of the sleeve, better training.
66	Product	2.4	Trauma to fetus	Poor view, slip of scalpel, malpositioning of fetus	Fetus needs to receive treatment, discontinuation of procedure	Discontinue of procedure	4	1	4		12	1	12	
67	Product	2.5	Sleeve tears	Material is too weak, too much pressure is loaded on the retractor, a sharp object touches the retractor	AAF can flow trough the sleeve, leakage inside abdominal cavity	Infection and leakage	3	2	6		3	2	6	An indication of the minimum and maximum required retraction on the retractors. Improved material that is puncture and tear resistant. Make use of impact-resistant materials.
68	Product	2.5	Too much pressure loaded onto retractor	Weak material, too much retraction, distance uterus to retractor connector to large	Sleeve tears, leakage in abdominal cavity, blood from incision site can accumulate	Air exposure, liquid drainage into abdomen	3	2	6		3	2	6	Make use of flexible, stiff, and impact-resistant materials. Proper dimensioning of retractor sleeve length.
69	Product	2.5	Puncture by e.g. scalpel	Weak material, poor view, poor skills, too narrow site for incision	Sleeve tears, leakage in abdominal cavity, blood from incision site can accumulate, tissue trauma needs to be repaired	Air exposure, liquid drainage into abdomen, tissue trauma	3	1	3		3	1	3	Clearly instruct that no sharp object may touch the sleeve of the retractor to prevent rupturing. Make use of impact resistant materials
70	Product	2.5	Retractor does not fit behind uterus wall	Poor design/dimensioning, wrongly positioned, incision too small	Retractor needs to be readjusted, perinate cannot pass through, obstructed view (no clear canal to uterus visible), trauma to perinate, leakage into abdominal cavity	Longer duration of procedure, trauma to perinate, leakage into abdominal cavity	4	2	8		12	2	24	Dimensioning should be adequate. Proper training of placement behind uterus wall. Mechanisms (ridge) to better attach interior ring behind wall might be needed.
71	Product	2.5	Interior uterus retractor ring is too large	Too wide diameter of retractor interior ring	Trauma to uterus tissue/perinate, too bulky material in uterus, decreased access to perinate	Longer duration of procedure, leakage of fluid from uterus around ring into abdominal cavity	3	2	6		3	2	6	Dimensioning should be adequate.
72	Product	2.5	Interior uterus retractor ring is too small	Too small diameter of retractor interior ring	Retractor does not hold tight to the uterus and by moving transferbag all liquid content might be emptied into abdominal cavity	Unstable ensemble of devices, leakage into abdominal cavity	4	2	8		12	2	24	Dimensioning should be adequate.
73	Product	2.6	Transferbag too heavy	Task time too long, transferbag contains too much liquid, not enough hands/support available	Operator drops transferbag	(Lethal) trauma, psychological damage to parents, ergonomic problems operators	4	4	16		12	4	48	Minimize duration how long the operator needs to hold the filled transferbag before delivery. Provide support during transfer procedure.
74	Product	2.6	Transferbag dropped	Not enough grip, too slippery environment, no support, too heavy, poor communication, stress, poor view	Trauma, leakage, stretching of UC	(Lethal) trauma, psychological damage to parents, ergonomic problems operators	4	2	8		12	2	24	The transferbag should be easy to hold and it must be prevented that the transferbag can slip out of the operators hand (e.g. handles, grip). Additional support necessary
75	Product	2.6	Transferbag too slippery	Slippery material, AAF leakage onto surgical space, no spots to hold the device	Operator might drop the transferbag or place too much pressure on perinate to grasp the bag	Trauma to the perinate, ergonomic/physical issues for the operator	3	4	12		3	4	12	The transferbag should be easy to hold and it must be prevented that the transferbag can slip out of the operators hand (e.g. handles, grip).
76	Product	2.6	Tubing too small	Poor dimensioning	Extended preparation, extended procedure duration, becoming too heavy to hold the transferbag	Ergonomic issues, risk of dropping too heavy transferbag	2	2	4		2	2	4	Improve tubing dimensions.
77	Product	2.6	Filling of AAF too slow	Tubing too small, too little pressure, leakage	Extended preparation, extended procedure duration, becoming too heavy to hold the transferbag	Ergonomic issues, risk of dropping too heavy transferbag	2	3	6		2	3	6	Improve tubing dimensions. Connection tube to transferbag should be able to attain enough pressure.
78	Product	2.6	AAF leakage	Poor dimensioning, poor material (not stress resistant), transferbag not properly attached to connector	Leakage, air entry into bag	Air exposure, rescue procedure	2	3	6		2	3	6	Ensure proper dimensioning and material that can hold the weight of all the liquid
79	Product	2.7	Clips break	Poor material choices, poor dimensioning, too much pressure, entrapment	Leakage, air entry into bag, perinate can become trapped between transferbag and retractor connector, clinicians divert attention to holding transferbag tight instead of care for perinate	Longer duration of procedure, leakage, air exposure	3	2	6		3	2	6	Adjust the clips in a way that the connection of the transferbag to the device ring is easy to attach and do not bother the operator. Proper material choices and dimensioning
80	Product	2.7	Entrapment of bulky sleeve/bag	Glove or sleeve is entrapped, bulky material, poor view	Ensemble of tools will not fit tight can lead to leakage of AAF	Procedure takes longer (readjusting of tools), tear of sleeve, leakage	2	3	6		2	3	6	Make sure that the device does not contain spots where something can get stuck. Improve material choices. Ensure enough duplicates of tools are available in case of tears.
81	Product	2.7	Too much force needed to use clip	Opertator applies too much pressure, (due to entrapment of material), stiff material of clip	Clips can break, too much force exerted on mothers abdomen	Ergonomics, maternal trauma	2	2	4		2	2	4	Clip construction should be well dimensioned to resist forces exerted.

82	Product	2.7	Transferbag not properly connected to retractor connector	Wrong dimensioning, entrapment of sleeve/glove/tissue between two parts	Leakage, too much force exerted on tools and thereby mother's abdomen	Readjusting tools (procedure takes longer), air exposure, rescue procedure	2	3	6	2	3	6	Ensure that the device ring fits tight inside the outer ring of the wound retractor to prevent movement or coming loose.
83	Product	2.7	Trapped air not released	Air vent is not working properly, vent is not releasing quick enough	Procedure takes longer, infant comes in contact with air	Procedure takes longer to allow air to be released (too long a procedure endangers the fetus because UC needs to be cannulated), air exposure	4	2	8	12	2	24	An indication to make clear how and where to vent out the air.
84	Product	2.7	AAF leakage	Transferbag not properly attached, poor material of transferbag (cannot handle weight)	Leakage, air entry into bag	Air exposure, rescue procedure	2	3	6	2	3	6	Ensure that the device is sealed watertight (fabrication and material choices).
85	Product	2.7	Air vent malfunction	Too small, poor material choices, wrong placement of vent on transferbag (design choice)	Procedure takes longer: new transferbag might need to be prepared and attached	Procedure takes longer to allow air to be released (too long a procedure endangers the fetus because UC needs to be cannulated), air exposure	3	2	6	3	2	6	Make sure that the excess air in the transferbag can easily be removed but that air from outside cannot enter the transferbag.
86	Product	2.7	Insufficient AAF supply	Tubing too small, too little pressure, leakage, AAF reservoir not sufficient	Extended preparation, extended procedure duration, becoming too heavy to hold the transferbag	Ergonomic issues, risk of dropping too heavy transferbag	4	2	8	12	2	24	Improve tubing dimensions. Connection tube to transferbag should be able to attain enough pressure. Improve AAF reservoir dimensions
87	Product	3.1	Perinate does not fit inside the bag	Poor material choices (too stiff), poor dimensioning	Extend duration of the procedure, too much force exerted to place infant in transferbag, breech presentation (perinate's head not in transferbag)	Trauma to perinate, air exposure if head is not brought into transferbag	3	3	9	3	3	9	Make sure that the perinate fits completely inside the transferbag but with enough space to clamp and close the bag. Different sizes of bags available.
88	Product	3.1	Cannot reach perinate using transferbag glove	Poor material choices (too stiff), poor dimensioning (glove should be elongated), not enough grip, not enough dexterity, poor view	Extended duration of the procedure, trauma to perinate if wrongly held	Trauma to perinate, extend duration, procedure cancelled	4	2	8	12	2	24	Ensure that the hand of the operator can smoothly enter the uterus (e.g. material, thickness, stiffness).
89	Product	3.1	Material too stiff	Poor material choices	Extended duration of the procedure, trauma to perinate if wrongly held, difficult to reach perinate, decreased dexterity	Trauma to perinate, extend duration, procedure cancelled	3	1	3	3	1	3	Make use of flexible, stiff, and impact-resistant materials.
90	Product	3.1	Material too slippery	Poor material choices, excessive fluid at the surgical site	Instability, transferbag could be dropped, perinate cannot be reached or slips out of hands gloves back into uterus	Trauma to perinate, extended duration, procedure cancelled	3	3	9	3	3	9	Make us of material that has enough grip on the surface.
91	Product	3.1	Insufficient hand dexterity	Poor material choices, poor dimensioning, too much pressure	Extended duration of the procedure, trauma to perinate if wrongly held, difficult to reach perinate, decreased dexterity	Trauma to perinate, extended duration, procedure cancelled	4	3	12	12	3	36	Ensure that the hand of the operator can smoothly enter the uterus (e.g. material, thickness, stiffness).
92	Product	3.1	Glove too small/large	Poor dimensioning, poor material choices (stiffness/flexibility)	material accumulation, risk of tearing the glove/transferbag, leakage, poor dexterity, difficult to reach perinate, extended duration of the procedure (transferbag might need to be changed	leakage, trauma to perinate, ergonomic/ physical issues for the operator	2	4	8	2	4	8	The glove should be comfortable for operators with small or large hands. Make multiple sizes of gloves available.
93	Product	3.1	Poor sight	Accumulation of material, transferbag opacity, positioning of mother	Difficult to reach perinate, extended duration before reaching perinate (asphyxia risk), trauma to perinate due to unintended place of holding hand	Trauma to perinate, ergonomic/physical issues operator, extended duration of procedure, procedure cancelled	3	3	9	3	3	9	Ensure that material is transparent enough, reduce risk of material accumulation
94	Product	3.2	No grip	Slippery material, no places to hold the device, excessive fluid at surgical site	Operator drops transferbag, extedned duration before reaching perinate, trauma to perinate (slips away and could hit parts of device)	Trauma to the perinate, ergonomic/physical issues for the operator	3	3	9	3	3	9	Make use of materials that have grip especially on the place where the operator holds the device.
95	Product	3.2	Transferbag dropped	Not enough grip, too slippery environment, no support, too heavy, poor communication, stress, poor view	Trauma, leakage, stretching of UC	(Lethal) trauma, psychological damage to parents, ergonomic problems operators	4	2	8	12	2	24	The transferbag should be easy to hold and it must be prevented that the transferbag can slip out of the operators hand (e.g. handles, grip). Additional support necessary
96	Product	3.2	Transferbag too heavy	Task time too long, transferbag contains too much liquid, not enough hands/support available	Operator drops transferbag	(Lethal) trauma, psychological damage to parents, ergonomic problems operators	4	4	16	12	4	48	Minimize duration how long the operator needs to hold the filled transferbag before delivery. Provide support during transfer procedure.
97	Product	3.2	Clips not detaching	Poor material choices, entrapment	Extended duration of the procedure, force is applied to maternal abdomen, transferbag would need to be cut if clips are not detaching at all	Trauma to mother, ergonomic problems operators, procedure cancelled	3	1	3	3	1	3	Adjust the clips in a way that the connection of the transferbag to the device ring is easy to attach and do not bother the operator.
98	Product	3.2	AAF leakage	Transferbag not properly (not quick enough) detached, poor material of transferbag (cannot handle weight)	Leakage, air entry into bag	Air exposure, rescue procedure	2	3	6	2	3	6	Ensure that the device is sealed watertight (fabrication and material choices).
99	Product	3.2	Hand clamped too tight/weak around UC	Poor view	Decreased UC blood flow, AAF can flow out and air can enter transferbag, leakage, perinate could fall out	Trauma to perinate	3	2	6	3	2	6	Moment of holding transferbag in this way should be minimized by offering support for transferbag.
100	Product	3.2	Hand is misplaced (while detaching transferbag)	Poor view	Decreased UC blood flow, AAF can flow out and air can enter transferbag, leakage, perinate could fall out	Trauma to perinate	1	2	2	1	2	2	Markers on tranfserbag to show where hand should be placed while detaching transferbag from retractor connector.
101	Product	4.1	Temperature drops	Poor material choices, not enough AAF (e.g. because of leakage), procedure takes too long	Hypothermia	Rescue procedure	4	2	8	12	2	24	Enough AAF should be supplied to the transferbag, enough AAF should be available in reservoir, measures to maintain perinate at temperature should be sufficient.
102	Product	4.1	Transferbag too heavy	Task time too long, transferbag contains too much liquid, not enough hands/support available	Operator drops transferbag	(Lethal) trauma, psychological damage to parents, ergonomic problems operators	4	4	16	12	4	48	Minimize duration how long the operator needs to hold the filled transferbag before delivery. Provide support during transfer procedure.
103	Product	4.1	No grip	Slippery material, no places to hold the device, excessive fluid at surgical site	Operator drops transferbag, extedned duration before reaching perinate, trauma to perinate (slips away and could hit parts of device)	Trauma to the perinate, ergonomic/physical issues for the operator	3	3	9	3	3	9	Make use of materials that have grip especially on the place where the operator holds the device.
104	Product	4.1	Transferbag instability during cannulation	Not enough grip, too slippery environment, no support, too heavy, poor view	Trauma, leakage, stretching of UC, cannula dislocation or rupture.	(Lethal) trauma, psychological damage to parents, ergonomic problems operators	4	2	8	12	2	24	The transferbag should be easy to hold and it must be prevented that the transferbag can slip out of the operators hand (e.g. handles, grip). Additional support necessary
