# Supplementary material for: From intra- to extra-uterine: early phase design of a transfer to extra-uterine life support through medical simulation
Source: Front Med Technol. 2024 Aug 20;6:1371447. doi: 10.3389/fmedt.2024.1371447 (PMC11368740; doi:10.3389/fmedt.2024.1371447)
Supplement: Supplementary file 5 [file Table1.docx]

Supplementary Material

# Supplementary Figures and Tables

## Supplementary Figures


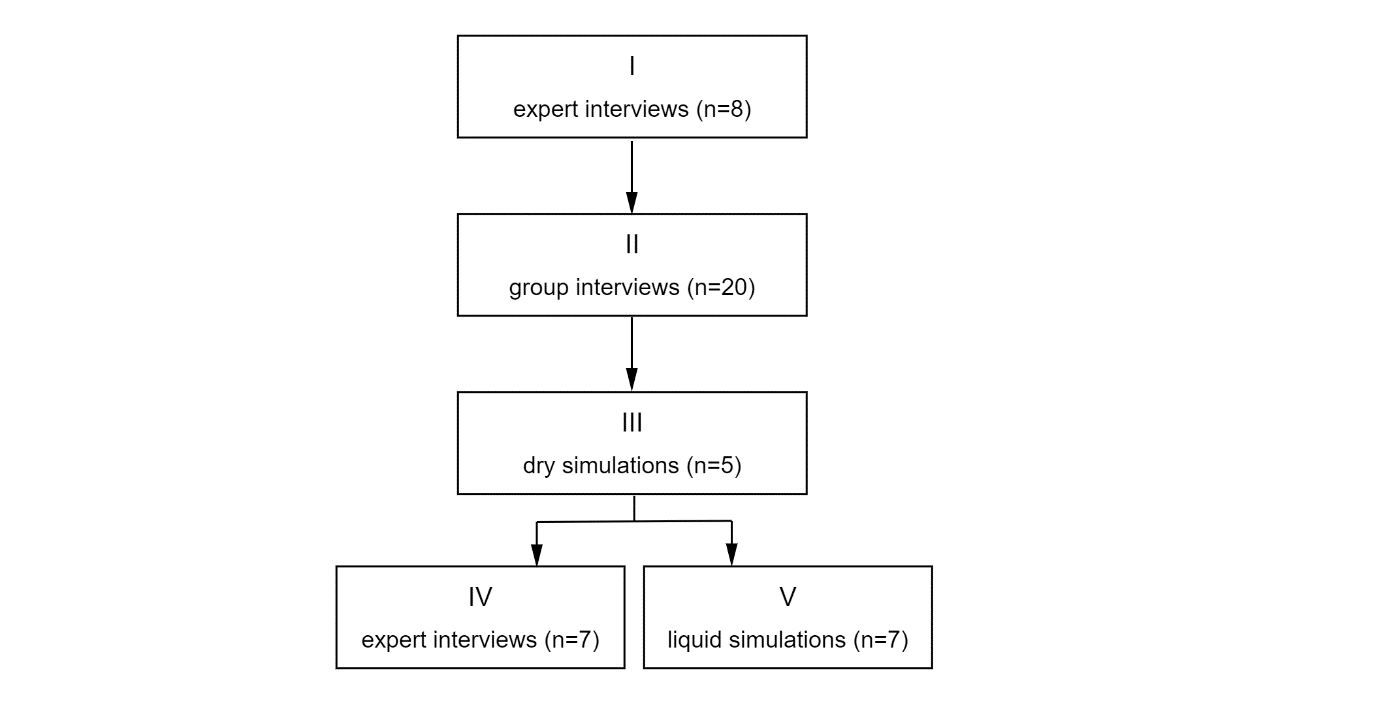


**Supplementary Figure 1.** Overview of the design phases.


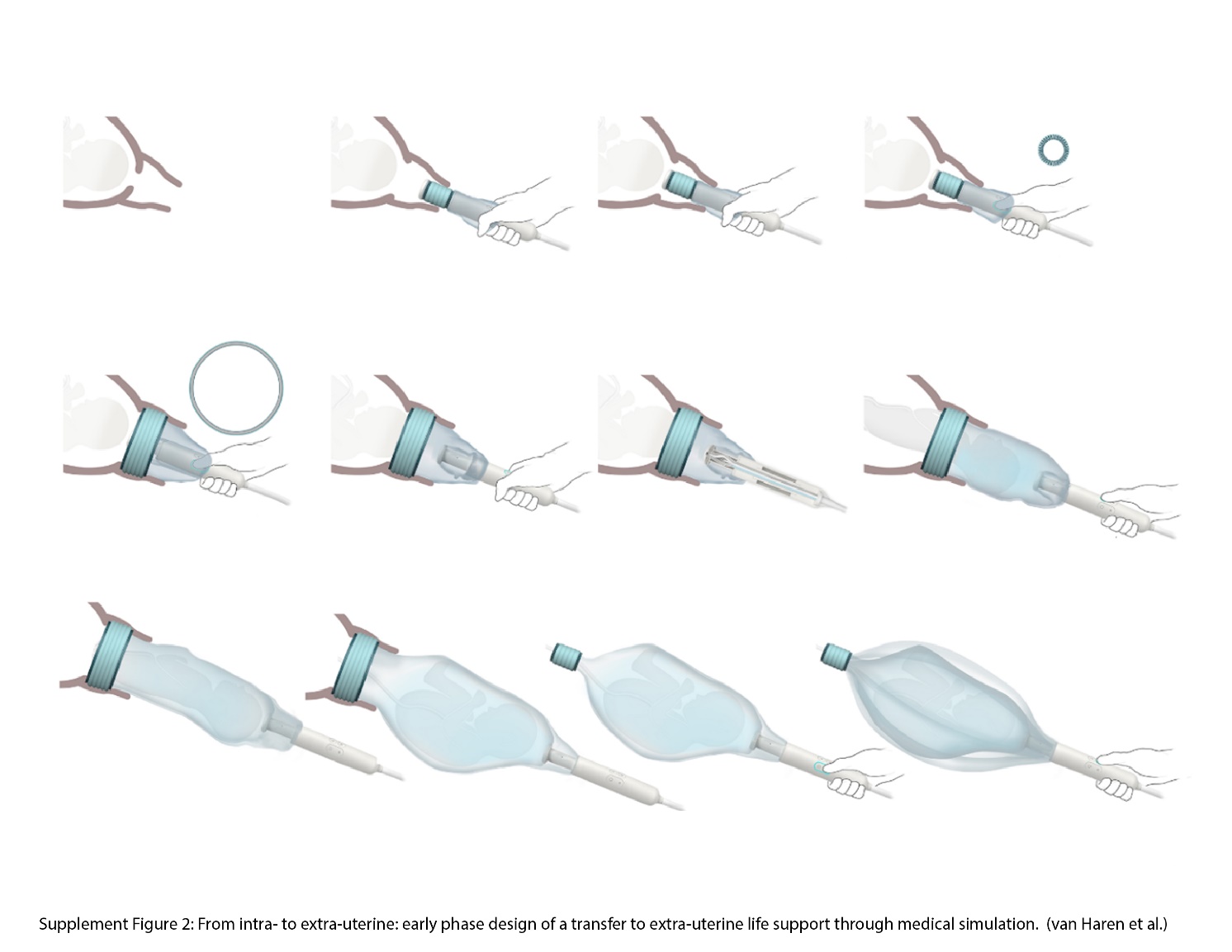


**Supplementary Figure 2.** First iteration VB transfer device prototype.

## Supplementary Tables

**Supplementary Table 1.** Requirements, dimensions, and parameters.

**I Product and shape**

| 1. | The wound retractor assures that the exposed wound edge from the abdominal incision is covered and protected from contaminants and that the tissue layers are tamponed to diminish blood accumulation |
| --- | --- |
| 2. | The retractors stay in form and position once they are set-up |
| 3. | A flexible, tear resistant cylindrical sleeve is attached between the inserted- and exterior ring |
| 4. | The retractor connector and the transferbag are connected over a labyrinth seal to ensure airtightness and to prevent leakage |
| 5. | The cross-sectional shape of the retractor connector includes a shape that prevents it from being partially or incorrectly connected to the exterior ring of the wound retractor |
| 6. | The retractor connector stays in place and has no friction during the procedure (can’t move upwards) |
| 7. | The transferbag must be detachable during the procedure if the uterus incision must be enlarged or to execute the rescue procedure |
| 8. | During the delivery the product should stay in place and friction should be prevented |
| 9. | The device contains no sharp edges to reduce the risk of infection, physical damage to the perinate and mother, and/or rupture of the transferbag |
| 10. | Trapped air in the transferbag can be released through a valve |
| 11. | The transferbag contains a tube connector for AAF supply + discharge to facilitate flow circulation to refresh the AAF and avoid cloudiness from blood that can impair visibility |

**II Material**

| 1. | Exterior rings of retractors: material must be compliant enough to allow the rings to be turned around its annular axis |
| --- | --- |
| 2. | The wound- and uterus retractor are sterile prior to use and can be distributed within a sterile packaging |
| 3. | The sheet of the wound- and uterus retractor is impermeable to fluids and bacteria, biocompatible and tear resistant and may additionally be coated with bactericidal or anti-inflammatory agents to further prevent infection or other complication during the surgical procedure. |
| 4. | The inner and outer ring are made of a material (f.e. Pellethane TPU, Lubrizol Co., Ohio, USA) that allows to maintain form after bending and rolling of the rings. |
| 5. | The cylindrical sheet connecting the rings is made of medical grade PU / TPU (Permali Ltd, Gloucester, UK), thin to avoid material accumulation in the incision, transparent, flexible, tear resistant and has sufficient strength when tamponing the wound edges and tissue. |
| 6. | The prototype of the retractor connector is made of an ABS like resin (Verowhite, Stratasys, Rehovot, Israël) and could be produced with a medical grade plastic such as polyethylene (PE) or thermoplastic polyurethane (TPU) (Ashby, 2005; Ashby, 2007). |
| 7. | The transferbag is made from medical grade TPU and has excellent tear and abrasion resistance, good microbiological and chemical resistance, excellent tensile strength and can be produced in different optical clarities, such as completely transparent, semi-transparent or opaque (Permali Ltd., Gloucester, UK). |

**III Dimensions and parameters**

| Part | Dimensions | Reference |
| --- | --- | --- |
| Uterus incision | Approx. 80 mm (size of fetal head) | Approximation |
| SurgiSleeve^TM^ wound retractor | 186 mm outer diameter  178 mm inner diameter  0.05 mm thickness | Medtronic, USA |
| Length of a 24-week old fetus | 300 mm  ± 200 mm (in fetal position) | MRI |
| Head of a 24 week old fetus | 222 mm = circumference (median)  70 mm = diameter | MRI |
| Minimum AAF volume (related to amniotic fluid level at 24 weeks of pregnancy) | 1000 mL | Brace & Wolf, 1989; Dubil & Magann, 2013 |
| Hand width (with thumb) | 116 mm = width (95^th^ percentile Dutch adults 20-60 mixed) | (Delft University of Technology Dined, 2021) |
| Transferbag | 0.10 mm thickness | Permali, UK |

| Gestational age (weeks) | Crown-rump (cm) | | Crown-heel (cm) | |
| --- | --- | --- | --- | --- |
|  | Mean | SD | Mean | SD |
| 24 | 21.3 | 1.8 | 30.4 | 2.4 |
| 28 | 25.4 | 2.0 | 36.1 | 2.7 |

|  | **min – max in cm** |
| --- | --- |
| Vagina lengths | 6.86 to 14.81 (Pendergrass et al, 1996) |
| Vagina width | 4.8 to 6.3 (Pendergrass et al, 1996) |
| Introital diameter | 2.39 to 6.45 (Pendergrass et al, 1996) |
| Cervical length during pregnancy | 3 to 4 (Nott et al, 2016; Egorov et al, 2020) |

|  | **(Range) Mean Value** | **SD** | **SI Unit** | **Source** |
| --- | --- | --- | --- | --- |
| Length fetus 24W old | 29.3 |  | cm | MRI |
| Weight fetus 24W old | 662 |  | g | Salomon et al, 2007 |
| Volume fetus 24W old | 539 |  | cm^3^ | MRI |
| Head circumference 24W old perinate | 213 |  | mm | MRI |
| Uterine volume including perinate and amniotic fluid 25W gestation | 1823 |  | ml | Geirsson, 1986 |
| Uterine volume including perinate and amniotic fluid 30W gestation | 2694 |  | ml | Geirsson, 1986 |
| Uterine volume with only amniotic fluid 24W gestation | 600 |  | ml | Gilbert, 2006 |
| Uterine volume with only amniotic fluid 28W gestation | 800 |  | ml | Underwood et al, 2005 |
| Cervical wall elasticity (pregnant women, avg 25.4W gestation) (Young's modulus) | (﻿4.9 - 58.6) 32 |  | kPa | Egorov et al, 2020 |
| Vagina wall elasticity (Young's modulus) | ﻿13.1 | 7.8 | kPa | Egorov et al, 2016 |
| ﻿Maximum circumferential stretch of levator muscle during childbirth | 248 |  | % | Sindhwani et al, 2017 |
| Vagina voluntary contraction pressure | (5.9-106.0) 41.8 | 18.4 | kPa | Egorov et al, 2018 |
| Vagina voluntary contraction force | (0.39-9.52) 3.20 | 1.94 | N | Egorov et al, 2018 |
| ﻿Vaginal maximum resistance force to insertion of device | (0.21-3.54) 1.09 | 0.7 | N | Egorov et al, 2018 |
| Spontaneous uterine contractions force at the pelvic inlet | 82 |  | N | Buhimschi et al, 2003 |
| Voluntary uterine contraction force on fetal head | 120 |  | N | Ashton-Miller et al, 2009 |
| Intrauterine pressure during maximal contractions | 19 |  | kPa | Ashton-Miller et al, 2009 |
| ﻿Maximum head to cervix force | (0.22-0.48) |  | N | Antonucci et al, 1997 |
| Maximum force of perinate head to pelvic floor muscles | 26 |  | N | Silva et al, 2015 |
| Maximum stress of perinate head to pelvic floor muscles | ﻿27.46 |  | Mpa | Krofta et al, 2017 |
| Largest LAM stretch ratio in  inferior direction | 250 |  | % | Krofta et al, 2017 |
| Potential tissue trauma at sustained compression | > 150 |  | kPa | Chandler et al, 2017 |
| Maximum traction force skull at birth (less chance of injury). | 450 |  | mm Hg | Unzila & Errol, 2009 |
| Time to first breath | 10.8 |  | sec | Vyas et al, 1986 |
| Placental delivery (duration after birth) | 8-9 |  | min | Magann et al, 2005 |

**Supplementary Table 2a.** Hierarchical Task Analysis CS.

| Task |  | Description | Task |  | Description | |  |
| --- | --- | --- | --- | --- | --- | --- | --- |
| 1 |  | Prepare | **2.6** |  | Fill transferbag | |  |
| 1.1 |  | Order device parts in sequence | **2.6.1** |  | Connect transferbag to AAF supply | |  |
| 2 |  | Insert transfer device | **2.6.2** |  | Hold transferbag | |  |
| 2.1 |  | Skin incision | **2.6.3** |  | Put 1 hand into integrated glove | |  |
| 2.1.1 |  | Hand over scalpel | **2.6.4** |  | Hold transferbag with 1 hand | |  |
| 2.1.2 |  | Take scalpel | **2.6.5** |  | Release hands from transferbag | |  |
| 2.1.3 |  | Perform incision up to uterus | **2.6.6** |  | Keep 1 hand inside transferbag | |  |
| 2.1.4 |  | Hand over scalpel | **2.6.7** |  | Fill transferbag with AAF | |  |
| 2.1.5 |  | Take scalpel | **2.6.8** |  | Hold transferbag with 2 hands | |  |
| 2.2 |  | Insert wound retractor | **2.6.9** |  | Stop holding but keep 1 hand inside | |  |
| 2.2.1 |  | Hand over wound retractor | **2.6.10** |  | Clamp transferbag with 1 hand | |  |
| 2.2.2 |  | Take wound retractor | **2.7** |  | Connect transferbag | |  |
| 2.2.3 |  | Insert wound retractor | **2.7.1** |  | Hold 1 hand inside and clamp tight with 1 hand | |  |
| 2.2.4 |  | Check bowel/tissue entrapment | **2.7.2** |  | Hold device with 2 hands | |  |
| 2.2.5 |  | Pull outer ring upwards | **2.7.3** |  |  | Hand over transferbag |  |
| 2.2.6 |  | Roll ring inwards to achieve desired retraction | **2.7.4** |  | Hold ring of transferbag with 2 hands | |  |
| 2.2.7 |  | Check bowel/tissue entrapment | **2.7.5** |  | Release hands from transferbag | |  |
| 2.3 |  | Insert retractor connector | **2.7.6** |  |  | Place transferbag in device ring | |
| 2.3.1 |  | Hand over retractor connector | **2.7.7** |  | Clamp bag to retractor connector | |  |
| 2.3.2 |  | Take retractor connector | **2.7.8** |  | Vent air out of transferbag | |  |
| 2.3.3 |  | Insert retractor connector | **2.7.9** |  | Remove non-dominant hand | |  |
| 2.3.4 |  | Check position | **3** |  | Deliver perinate | |  |
| 2.4 |  | Uterus incision | **3.1** |  | Deliver perinate | |  |
| 2.4.1 |  | Hand over scalpel | **3.1.1** |  | Hold transfer device in place | |  |
| 2.4.2 |  | Take scalpel | **3.1.2** |  | Hand into uterus to deliver perinate | |  |
| 2.4.3 |  | Perform uterus incision | **3.1.3** |  | Grab perinate | |  |
| 2.4.4 |  | Handover scalpel | **3.1.4** |  | Deliver perinate into end of transferbag | |  |
| 2.4.5 |  | Take scalpel | **3.1.5** |  | Clamp bag with non-dominant hand | |  |
| 2.5 |  | Insert uterus retractor | **3.2** |  | Detach transfer device | |  |
| 2.5.1 |  | Hand over uterus retractor | **3.2.1** |  | Detach the clips | |  |
| 2.5.2 |  | Take uterus retractor | **3.2.2** |  | Detach transferbag | |  |
| 2.5.3 |  | Insert uterus retractor | **4** |  | Cannulation | |  |
| 2.5.4 |  | Check perinate/tissue entrapment | **4.1** |  | Stably place transferbag | |  |
| 2.5.5 |  | Pull outer ring upwards | **4.1.1** |  | Prepare platform | |  |
| 2.5.6 |  | Roll outward to achieved desire retraction | **4.1.2** |  | Keep hand inside to support perinate | |  |
| 2.5.7 |  | Check perinate/tissue entrapment | **4.1.3** |  | Keep hand clamped around bag | |  |
| 2.5.8 |  | Place outer ring inside retractor connector | **4.1.4** |  | Hold and support ring of transferbag | |  |
| 2.5.9 |  | Fill incision site with AAF | **4.1.5** |  | Place transferbag ring in holder | |  |
| 2.5.9.1 |  | Hand over AAF | **4.1.6** |  | Pull out hand from integrated glove | |  |
| 2.5.9.2 |  | Take AAF to fill | **4.2** |  | Assess perinate | |  |
|  |  |  | **4.3** |  | Cannulation | |  |
|  |  |  | **5** |  | Maternal aftercare | |  |
|  |  |  | **5.1** |  | Removal of retractors and connector | |  |
|  |  |  | **5.2** |  | Placenta delivery | |  |

| Perinatologist 1 | Perinatologist 2 | Surgical assistant |
| --- | --- | --- |

**Supplementary Table 2b.** Hierarchical Task Analysis VB.

| Task number |  | Task | Task number |  | Task |  |
| --- | --- | --- | --- | --- | --- | --- |
| 1 |  | Prepare | **2.4** |  | Connect transferbag |  |
| 1.1 |  | Order device parts in sequence | **2.4.1** |  | Hold 1 hand in integrated glove and clamp tight with 1 hand |  |
| 1.2 |  | Determine cervical dilation | **2.4.2** |  | Hold transferbag with 2 hands |  |
| 1.3 |  | Cleaning of birth canal | **2.4.3** |  | Connect bag to retractor with clips |  |
| 2 |  | Use transfer device | **2.4.4** |  | Fill additional AAF (into birth canal) |  |
| 2.1 |  | Insert retractor | **2.4.5** |  | Vent air out of transferbag |  |
| 2.1.1 |  | Hand over retractor | **2.4.6** |  | Remove non-dominant hand |  |
| 2.1.2 |  | Take retractor | **3** |  | Deliver perinate |  |
| 2.1.3 |  | Fold retractor | **3.1** |  | Deliver perinate |  |
| 2.1.4 |  | Place retractor in birth canal | **3.1.1** |  | Hold transfer device in place |  |
| 2.1.5 |  | Confirm position retractor | **3.1.2** |  | Hand towards birth canal to deliver perinate |  |
| 2.2 |  | Inflate retractor | **3.1.3** |  | Grab perinate |  |
| 2.2.1 |  | Take bellow | **3.1.4** |  | Deliver perinate into end of transferbag |  |
| 2.2.2 |  | Inflate bellow to achieve desired retraction | **3.1.5** |  | Clamp bag with non-dominant hand |  |
| 2.2.3 |  | Re-confirming position retractor | **3.2** |  | Detach transfer device |  |
| 2.2.4 |  | Determine cervical dilation | **3.2.1** |  | Detach the clips |  |
| 2.2.5 |  | Use amniotomy hook to rupture membranes (optional) | **3.2.2** |  | Detach transferbag from retractor |  |
| 2.3 |  | Fill transferbag | **3.2.3** |  | Deflate retractor |  |
| 2.3.1 |  | Connect transferbag to AAF supply | **4** |  | Cannulation |  |
| 2.3.2 |  | Hold transferbag | **4.1** |  | Stably place transferbag | |
| 2.3.3 |  | Put 1 hand into integrated glove | **4.1.1** |  | Prepare platform |  |
| 2.3.4 |  | Hold transferbag with 1 hand | **4.1.2** |  | Keep hand inside integrated glove to support perinate |  |
| 2.3.5 |  | Release hands from transferbag | **4.1.3** |  | Keep hand clamped around bag |  |
| 2.3.6 |  | Keep 1 hand inside transferbag | **4.1.4** |  | Hold and support ring of transferbag |  |
| 2.3.7 |  | Fill transferbag with AAF | **4.1.5** |  | Place transferbag ring in holder |  |
| 2.3.9 |  | Stop holding but keep 1 hand inside | **4.1.6** |  | Pull out hand from integrated glove |  |
| 2.3.10 |  | Clamp transferbag with 1 hand | **4.2** |  | Assess perinate |  |
|  |  |  | **4.3** |  | Cannulation |  |
|  |  |  | **5** |  | Maternal aftercare |  |
|  |  |  | **5.1** |  | Removal of retractor |  |
|  |  |  | **5.2** |  | Placenta delivery |  |

| Perinatologist 1 | Perinatologist 2 | Surgical assistant |
| --- | --- | --- |

**Supplementary Table 3a.** Fault Tree Analysis VB.

[Separate pdf file]

**Supplementary Table 3b.** Fault Tree Analysis CS.

[Separate pdf file]

**Supplementary Table 4a.** Risk assessment matrix and FMEA for VB.

[Separate pdf file]

**Supplement Table 4b.** Risk assessment matrix and FMEA for CS

[Separate pdf file]
